# Increased gene dosage of *RFWD2* causes autistic-like behaviors and aberrant synaptic formation and function in mice

**DOI:** 10.1038/s41380-024-02515-7

**Published:** 2024-03-19

**Authors:** Yong-Xia Li, Zhi-Nei Tan, Xu-Hui Li, Boyu Ma, Frank Adu Nti, Xiao-Qiang Lv, Zhen-Jun Tian, Riqiang Yan, Heng-Ye Man, Xin-Ming Ma

**Affiliations:** 1 College of Life Sciences, Shaanxi Normal University, Xi’an, China; 2 Center for Neuron and Disease, Frontier Institutes of Science and Technology, Xi’an Jiaotong University, Xi’an, China; 3 Department of Oral and Maxillofacial Surgery, University of Alabama at Birmingham, Birmingham, AL USA; 4 Institute of Sports Biology, College of Physical Education, Shaanxi Normal University, Xi’an, China; 5 Department of Neuroscience, University of Connecticut Health, Farmington, CT USA; 6 Department of Biology, Boston University, Boston, MA USA

**Keywords:** Autism spectrum disorders, Psychology

## Abstract

Autism spectrum disorder (ASD) is a neurodevelopmental disorder characterized by impaired social interactions, communication deficits and repetitive behaviors. A study of autistic human subjects has identified *RFWD2* as a susceptibility gene for autism, and autistic patients have 3 copies of the *RFWD2* gene. The role of RFWD2 as an E3 ligase in neuronal functions, and its contribution to the pathophysiology of ASD, remain unknown. We generated *RFWD2* knockin mice to model the human autistic condition of high gene dosage of *RFWD2*. We found that heterozygous knockin (*Rfwd2*^+/−^) male mice exhibited the core symptoms of autism. *Rfwd2*^+/−^ male mice showed deficits in social interaction and communication, increased repetitive and anxiety-like behavior, and spatial memory deficits, whereas *Rfwd2*^+/−^ female mice showed subtle deficits in social communication and spatial memory but were normal in anxiety-like, repetitive, and social behaviors. These autistic-like behaviors in males were accompanied by a reduction in dendritic spine density and abnormal synaptic function on layer II/III pyramidal neurons in the prelimbic area of the medial prefrontal cortex (mPFC), as well as decreased expression of synaptic proteins. Impaired social behaviors in *Rfwd2*^+/−^ male mice were rescued by the expression of ETV5, one of the major substrates of RFWD2, in the mPFC. These findings indicate an important role of RFWD2 in the pathogenesis of autism.

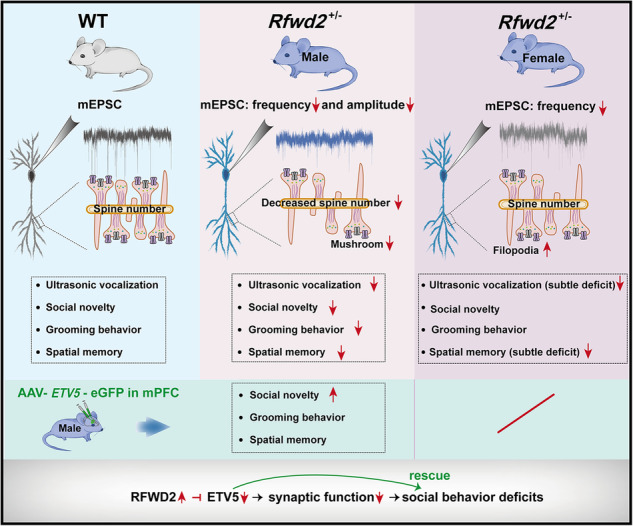

## Introduction

Autism Spectrum Disorder (ASD), a neurodevelopmental disorder, is characterized by deficits in social behaviors, communication, and repetitive behaviors. These core symptoms are often comorbid with anxiety [1] and memory deficits [2]. Approximately 1 in 36 (2.8%) of 8-year-old children in the United States have been diagnosed with ASDs [3, 4]. ASDs are more common in males than females [5] and result from interactions between genetic, environmental, and immunologic factors [6–8]. Genetic variation and chromosomal deletions/duplications are the main factors contributing to ASDs [9–11]. A large number of candidate genes for ASDs have been identified [12], including well-studied genes such as *SHANK* [13], *NEXMIF* [14], *NLGN3* [15], and *MeCP2* [16]. A prominent cluster of autism risk genes are components of the ubiquitin-proteasome system (UPS), which is involved in controlling proteostasis in the brain [17].

Protein homeostasis in cells is tightly controlled by the UPS, which selectively degrades its substrates via the sequential function of three enzymes: E1, E2, and E3 ubiquitin ligase [18]. Dysregulation of the UPS, particularly of E3 ligase such as UBE3A and PARK2, plays an important role in ASD and other neurological disorders [19–22]. Consistent with this, the E3 ligase Ring Finger and WD Domain 2 (*RFWD2*, also known as *COP*1) has been implicated in the etiology of ASD through copy number variations (CNVs) [11]. The human *RFWD2* gene is located at the chromosomal position 1q25 and is highly conserved among vertebrates. In mammals, RFWD2 is expressed in the brain and is localized to both the nucleus and cytoplasm [23]. While the involvement of RFWD2 in neuroinflammation [24] and neurodegeneration [25] has been studied. However, the neurobiological functions of RFWD2 in the brain remain largely unknown. The major ubiquitination substrates of RFWD2 include the ETS transcription factors (ETV1, ETV4, and ETV5) [25], of which ETV5 has been shown to play a role in hippocampal spine formation and synaptic function and is associated with social and cognitive functions [26].

To recapitulate the genetic condition of human *RFWD2* ASD, we generated *RFWD2* conditional knockin mice in which the heterozygous *Rfwd2*^+/−^ mice contain three copies of the *RFWD2* gene. We found that *Rfwd2*^+/−^ male mice showed autistic-like behaviors characterized by social behavioral deficits, impaired communication, repetitive behaviors, anxiety-like behaviors, and spatial memory deficits. In contrast, *Rfwd2*^+/−^ female mice showed only subtle deficits in communication and short-term spatial memory. The medial prefrontal cortex (mPFC) includes the prelimbic (PrL), infralimbic, and anterior cingulate cortices in rodents. PrL pyramidal neurons, which project to various brain areas, play important roles in emotion, cognition, and social behavior [27–29]; Dysfunction of the PrL neural circuit has been shown to be associated with autistic-like behaviors [29, 30]. Therefore, we aimed to understand the role of RFWD2 in neuronal development and function in the mPFC region. *Rfwd2*^+/−^ male mice showed a decrease in spine density and impaired synaptic functions on PrL cortical neurons, and decreased levels of synaptic proteins including Vglut1 and NR2B in the mPFC. More importantly, restoration of the RFWD2 target ETV5 in the mPFC rescued autistic-like behaviors in *Rfwd2*^+/−^ male mice. Our findings demonstrate that an increased dosage of RFWD2 contributes to the autistic phenotype and provide insights into the mechanisms underlying RFWD2-related ASD.

## Methods

All animal experimental procedures were approved by the Animal Care and Use Committee of Shaanxi Normal University. All animals were housed under a 12/12 h light/dark cycle (7:00 AM to 7:00 PM) at an appropriate temperature (23 °C ± 1 °C) and relative humidity (55% ± 10%) with free access to food and water. The study was conducted in accordance with the ethical principles of animal use and care.

### Generation of *Rfwd2* mutation mice

**Fig. 1:**
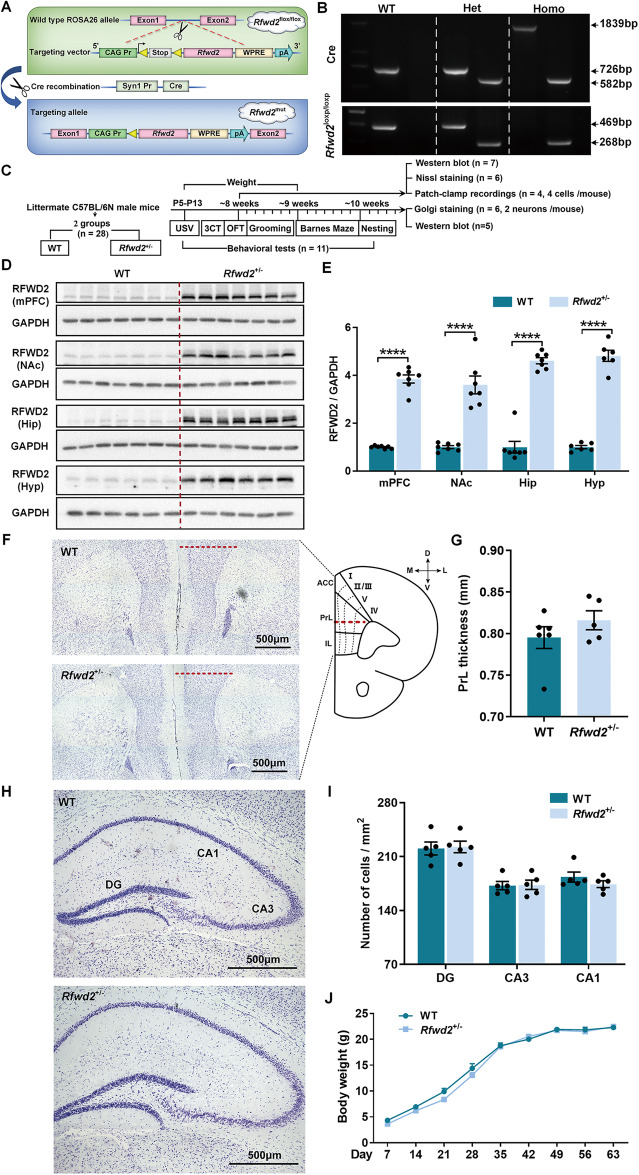
Targeting strategy and generation of *Rfwd2*^+/−^ mice. **A** Schematic representation of the gene targeting strategy. Homozygous *Rfwd2* knockin-floxed (*Rfwd2*^flox/flox^) mice, in which Rfwd2 is controlled by the flox-stop-flox cassette, were generated by introducing the targeting vector CAG-Pr-loxp-Stop-loxp-Rfwd2--WPRE-pA sequences into the wild-type (WT) ROSA26 allele of the C57/B6N mouse. The Cre recombinase protein driven by the synapsin I promoter mediates the deletion of the stop cassette flanked by two loxp sites (yellow triangles). Heterozygous *Rfwd2* knockin mutant heterozygous (*Rfwd2*^*+/−*^) mice expressing Cre and 3 copies of *Rfwd2*, which were generated by crossing *Rfwd2*^flox/flox^ mice with Syn1-Cre mice, were intercrossed to generate *Rfwd2*^*+/−*^, homozygous (homo) *Rfwd2*^*+/+*^ mice and wild-type (WT) littermates. **B** Genotyping of *Rfwd2*^+/−^ mice by PCR. **C** Experimental timeline. **D** Western blot analysis of RFWD2 protein in the medial prefrontal cortex (mPFC), nucleus accumbens (NAc), hippocampus (Hip), and hypothalamus (Hyp) of *Rfwd2*^+/−^ male mice and their WT littermates. **E** Levels of RFWD2 protein were increased in *Rfwd2*^+/−^ mice compared to WT littermates. **F**–**I** Nissl staining of cortical brain slices from WT and *Rfwd2*^+/−^mice at P60 showed no significant differences in mPFC thickness or cell number in the dentate gyrus (DG), or cornu ammonis area 3 (CA3), and cornu ammonis area 1 (CA1). **J** There was no significant difference in body weight between *Rfwd2*^+/−^ male mice and WT male littermates. **J** Two-way RM ANOVA, others with two-population Student’s t-test. Data are presented as mean ± SEM. ****p < 0.0001. *n* = 6–7.

*Rfwd2* conditional knockin-floxed mice were generated using a CRISPR/Cas9-based extreme genome editing (EGE) method in the C57BL/6 N strain (Fig. 1A). Synapsin1 (Syn1)-Cre mice in the C57BL/6 N background and *Rfwd2* conditional knockin-floxed mice were backcrossed onto C57BL/6 N mice for two generations prior to any experiments, respectively. Heterozygous *Rfwd2* knockin mutant (*Rfwd2*^*+/−*^*)* mice expressing Cre and 3 copies of RFWD2, which were generated by crossing *Rfwd2* knockin-floxed mice with Syn1-Cre mice, were intercrossed to generate heterozygous *Rfwd2*^*+/−*^ mice, homozygous Rfwd2 mutant (*Rfwd2*^*+/+*^) mice expressing Cre and 4 copies of RFWD2, and wild-type (WT) littermates expressing 2 copies of RFWD2. *Rfwd2*^*+/−*^ male and female mice, and their WT littermates of both sexes were used in this study. See supplementary methods.

### Behavioral tests

Ultrasonic vocalization (USV) recordings were performed on postnatal (P) days 5, 7 and 9 (P5, P7, P9), and other behavioral tests were performed on the same mice at 2 months of age. All animals were coded, and behavioral tests were performed by investigators blinded to genotype and treatment.

### Open field test (OFT)

The OFT has been widely used to analyze locomotor activity, anxiety-like, and repetitive behaviors in rodent ASD [14, 29, 30]. See supplementary methods for further details.

### Ultrasonic vocalization (USV) recording

The USV is used by rodents to communicate with each other and is a useful tool for assessing social communication; impaired communication is a hallmark of behavioral deficits in ASD [14] and underlies the neural basis of social behavior [31]. See supplementary method for further details.

### Three chamber social test

This test was performed as described in our previous report [14]. See supplementary methods for further details.

### Nest building behavior

Mice are highly motivated to build nests. Normal nesting behavior is an indicator of good performance, well-being, and healthy functioning [32]. See supplementary methods for further details.

### Barnes maze test (BMT)

The BMT was used to investigate spatial learning and memory and was performed as described in our previous study [33]. See supplementary methods for further details.

### Golgi staining

Dendritic spines on the PrL layer II/III pyramidal neurons of male mice were visualized by Golgi impregnation 24 h after behavioral testing as described in our previous study [34]. See supplementary methods for further details.

### DiOlistic labeling of dendritic spines

Dendritic spines on PrL layer II/III pyramidal neurons of female mice were visualized by filling dendrites with DiI (Invitrogen, Carlsbad, CA) using a gene gun as described in our previous study [35]. See supplementary methods for further details.

### Dendritic spine analysis

For quantification of spine density and types, collapsed z-stack images of the second-order segment on the apical dendrite of layer II/III pyramidal neurons were coded. Quantification of spine density was performed blindly using MetaMorph as described in our previous studies [34, 35]. See supplementary methods for further details.

### Western blot

Western blot experiments were performed as previously described [33]. See supplementary methods for further details.

### Nissl staining and cell analysis

See supplementary methods for details.

### Electrophysiological recordings on mPFC brain slices

Patch-clamp recordings were performed on PrL layer II/III pyramidal neurons. See supplementary methods for further details.

### AAV injection

The mPFC of *Rfwd2*^*+/−*^ male mice and WT mice at 8 weeks of age were bilaterally injected with AAV9-EF1α-*ETV5*-EGFP (ETV5) or control AAV9-EF1α-EGFP (vehicle control), respectively, using standard mouse stereotaxic methods. See supplementary methods for further details.

### Determination of the estrous cycle

Behavior in the female mouse is not affected by the estrous cycle [36]. Spine density on layer 5 pyramidal neurons of the mouse somatosensory cortex is stable throughout the estrous cycle [37, 38]. In addition, spine density on layer 3 pyramidal neurons of the neocortex [39], and layer 5 pyramidal neurons of both the mPFC [40] and the motor cortex [41] does not change during the estrous cycle in rats. These results suggest that the spine density of the mPFC pyramidal neurons in female mice is stable during the estrous cycle. There is a difference in basal glutamatergic synaptic transmission on mPFC pyramidal neurons between the proestrus and diestrus stages in female mice [42], for example, AMPA-mediated no NMDA EPSC frequency, but not amplitude on these neurons is lower in the proestrus than in the diestrus stage [42]. Therefore, female mice at the same proestrus stage were used for electrophysiology. Other experiments in females were performed as in other similar studies in female mice without the estrous cycle determination [4, 43, 44]. See supplementary methods for further details.

### Statistical analyses

All statistical tests were performed by investigators blinded to genotype, and/or groups using GraphPad Prism software (ver. 8.2; GraphPad Software, CA, United States). Values are expressed as mean ± SEM. Inspection of the data did not reveal any deviation from normality. Variances between groups were similar. Animals were randomly assigned to the experimental groups. The sample size (*n*) for different experiments is indicated in each figure legend and was determined according to the preliminary results and the standards generally used in the field [29, 35, 45]. Results were analyzed by two population Student’s *t*-test, or two-way ANOVA followed by post hoc Bonferroni’s test, or two-way repeated measures (RM) ANOVA followed by post hoc Sidak’s test, as indicated in the figure legends. Correlations were analyzed using Pearson correlation analysis. All variables are treatments/conditions, and all are all fixed. All the F- and *p*-values of T-test, two-way ANOVA and three-way ANOVA are reported in Supplementary Table S1. A *p*-value ≤ 0.05 was considered to be significant. Box-and-whisker plots are used to present the data distribution in the figures.

## Results

### Generation of *Rfwd2*^*+/−*^ mice

To establish a transgenic mouse line of conditional, neuronal specific overexpression of Rfwd2, we first generated a mouse line that contains an extra *Rfwd2* gene under control of a flox-stop-flox cassette (*Rfwd2*^flox/flox^ mice) (Fig. 1A). Heterozygous *Rfwd2* knockin mutant mice (*Rfwd2*^+/−^) expressing 3 copies of *Rfwd2* gene were then generated by crossing *Rfwd2*^flox/flox^ and Synapsin1 (*Syn1*)-*cre* mice (Fig. 1A). For *Syn1-cre* mice, the PCR products of WT, heterozygous (het), and homozygous (homo) mice were 726 bp, 726 bp & 582 bp, and 1839bp & 582 bp, respectively. For *Rfwd2*^flox/flox^ mice, the PCR products from WT, het *Rfwd2*^+/loxp^, and homo *Rfwd2*^loxp/loxp^ mice were 469 bp, 469 bp & 268 bp, and 268 bp, respectively (Fig. 1B). *Rfwd2*^+/−^ mice with expected higher levels of RFWD2 expression, together with WT littermates were used for the behavioral tests (Fig. 1C) and molecular studies.

As expected, RFWD2 protein levels were increased in the nucleus accumbens (NAc) (*p* < 0.0001), mPFC (*p* < 0.0001), hippocampus (*p* < 0.0001), and hypothalamus (*p* < 0.0001) of *Rfwd2*^+/−^ male mice compared to WT littermates (Fig. 1D, E). *Rfwd2*^+/−^ mice showed no differences in mPFC thickness or in cell number in the hippocampal DG, CA1 and CA3 regions compared to WT littermates (Fig. 1F–I). There were also no significant differences in body weight gain between WT and *Rfwd2*^+/−^ mice during postnatal development (Fig. 1J). These results demonstrate that *Rfwd2*^+/−^ male mice have normal gross morphology in the hippocampus and mPFC.

### *Rfwd2*^+/−^ male mice show deficits in communication and social behavior

**Fig. 2:**
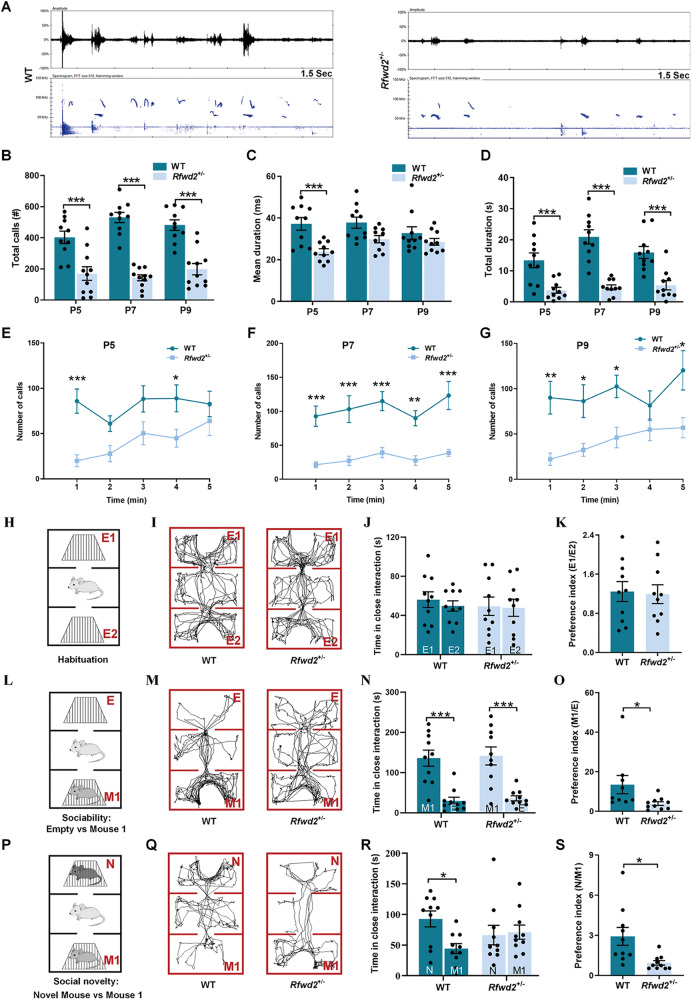
*Rfwd2*^+/−^ male mice showed a communication deficit in ultrasonic vocalization recording and impaired social behavior in the 3-chamber test. **A** Representative images of vocalizations recorded from *Rfwd2*^+/−^ mice and WT littermates on postnatal (P) days 5, 7, and 9. Top, oscillogram signals. Bottom, corresponding spectrogram signals; all are 1.5 s long. **B** Quantification of the call rate revealed a decrease in the total number of calls made by *Rfwd2*^+/−^ mice compared to WT littermates at P5, 7, and 9. **C**, **D** Mean (**C**) and total duration (**D**) of calls during the 5-min recording period at P5, P7 and P9 in *Rfwd2*^+/−^ mice and WT littermates. **E**–**G** Mean number of calls emitted during the 5 min recording period at P5, P7, and P9. **H** Habituation phase: *Rfwd2*^+/−^ and WT mice were allowed to explore the 3 chambers (center and the other two side chambers, containing two empty holding cells). **I**–**K** *Rfwd2*^+/−^ and WT littermates spent similar amount of time in the two empty chambers and showed equal preference (E1/E2 ratio). **L** In the sociability test, *Rfwd2*^+/−^ and WT mice were allowed to explore the 3 chambers, with one side chamber containing a novel mouse 1 (M1) and the other side chamber containing an empty (**E**) holding cell. **N** *Rfwd2*^+/−^ mice and WT littermates displayed a reduced preference for the side chamber containing the empty holding cell than the chamber with the M1 mouse. **O** *Rfwd2*^+/−^ mice showed a decreased preference (M1/E ratio) for mouse 1 compared to WT littermates. **P** In the social novelty test, a novel mouse (**N**) was placed in the opposite chamber, and M1 became a familiar mouse. *Rfwd2*^+/−^ and WT mice were allowed to explore all the chambers. **R** *Rfwd2*^+/−^ mice spent less time interacting with the novel mouse than the WT littermates and showed a decrease in preference (N/M1 ratio) for novel mice compared to WT littermates (**S**). Representative traces of ‘Empty 1-Empty 2’ (**I**) ‘Empty-Mouse 1’ (**M**) and ‘Novel mouse–Mouse 1’ (**Q**) for *Rfwd2*^+/−^ mice and WT littermates. Data are shown as mean ± SEM. **B**–**G** Two-way repeated-measures ANOVA followed by Sidak’s test; (**J**, **N**, **R**), two-way ANOVA followed by Bonferroni’s test; (**K**, **O**, **S**), two-population Student’s t-test. *p < 0.05, **p < 0.01, ***p < 0.001. *n* = 9, 10.

Ultrasonic vocalizations (USVs) in pups are important for mother-infant social interaction [46], and alterations in sociability are typical deficits often observed in ASD [47]. Isolation-induced USVs are commonly used to assess communication deficits in rodent models of ASD. Therefore, call emissions (isolation-induced USVs) were recorded for 5 min and analyzed in pups on P5, P7, and P9. The spectrogram and oscillogram from P5 are shown in Fig. 2A. *Rfwd2*^flox/flox^ and *syn1-cre* mice showed normal communication and normal social behavior [Supplementary Figs. (SFs) 2, 3]. Two-way RM ANOVA followed by Sidak’s test showed a significant difference between WT and *Rfwd2*^+/−^ mice in total calls (*F*_1,18_ = 135.80, *p* < 0.001, Fig. 2B). *Rfwd2*^+/−^ mice also showed significant changes in mean duration (*F*_1,18_ = 18.97, *p* < 0.001, Fig. 2C) and total duration (*F*_1,18_ = 96.33, *p* < 0.001, Fig. 2D). When USVs were analyzed per minute of the 5 min recordings, *Rfwd2*^+/−^ mice showed a significant decrease in the number of calls at P5 (*F*_1,16_ = 10.06, *p* < 0.01, Fig. 2E), P7 (*F*_1,16_ = 82.24, *p* < 0.001, Fig. 2F), and P9 (*F*_1,16_ = 36.75, *p* < 0.001, Fig. 2G).

The three-chamber tests were performed at P60 to evaluate sociability and social novelty. During the habituation period, *Rfwd2*^+/−^ mice and their WT littermates showed similar preferences for the two empty cages (Fig. 2H–K). During the sociability session, both *Rfwd2*^+/−^ and WT mice spent more time with the novel mouse 1 (M1) than with the empty chamber (*p* < 0.001, Fig. 2L–N), but *Rfwd2*^+/−^ mice showed a significant decrease in the preference index for novel mouse M1 compared to WT littermates (*p* < 0.05, Fig. 2O). In the social novelty test, a novel mouse was introduced into the empty chamber in the presence of the previously placed M1 mouse. WT littermates showed more interaction with the novel mouse N than with the familiar mouse M1 (*p* < 0.05, Fig. 2P–R). In contrast, *Rfwd2*^*+/−*^ mice spent the same amount of time interacting with both mice (Fig. 2P–R). *Rfwd2*^*+/−*^ mice showed a significant decrease in the preference index for the novel mouse compared to WT littermates (*p* = 0.01, Fig. 2S). Thus, *Rfwd2*^+/−^ mice showed impairments in both social preference and social novelty.

### *Rfwd2*^+/−^ male mice show repetitive behaviors, anxiety-like behaviors, and impaired nesting behavior

**Fig. 3:**
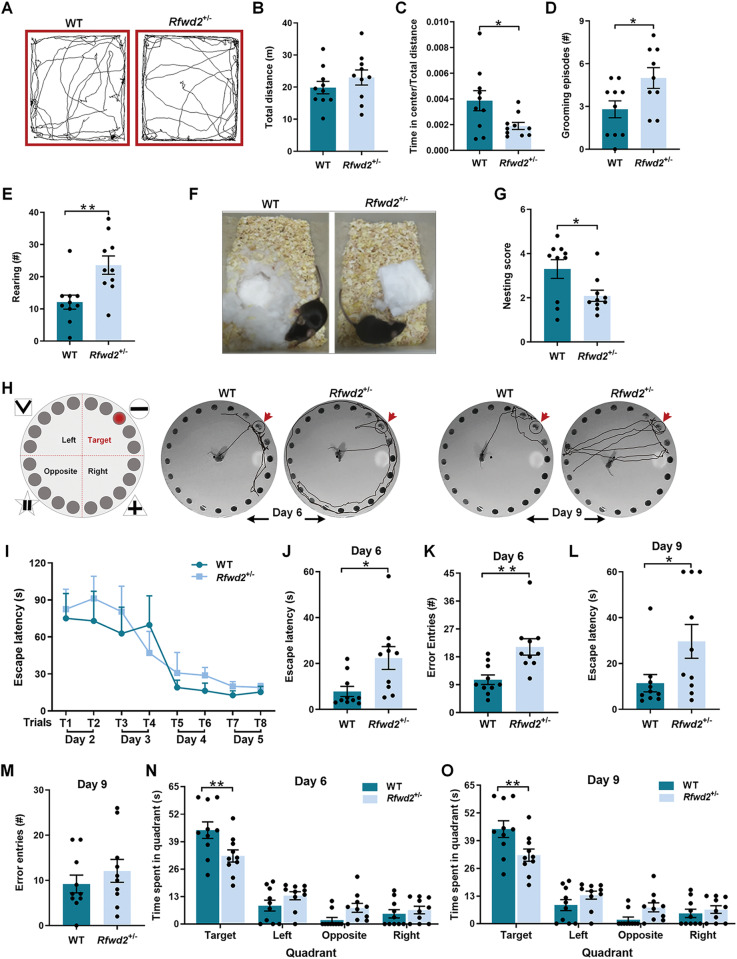
*Rfwd2*^+/−^ male mice showed normal locomotor activity, anxiety-like behavior, increased rearing and grooming, and impaired spatial memory. **A** Representative tracks of WT and *Rfwd2*^+/−^ mice in the open field test. *Rfwd2*^+/−^ mice showed normal locomotion (**B**), a decrease in time spent in the center (**C**), an increase in grooming (**D**), and an increase in rearing (**E**) in the open field. **F** Representative images of nesting between WT and *Rfwd2*^+/−^ mice. **G** *Rfwd2*^+/−^ mice showed a significant impairment in nesting behavior. **H**–**O** *Rfwd2*^+/−^ mice showed spatial memory deficits in the Barnes maze test. **H** Schematic diagram of the Barnes maze test, and tracking plots of the probe trials on days 6 and 9 in *Rfwd2*^+/−^ mice and WT littermates. **I** Escape latency during the training trials on days 2–5. Latency to find the target hole was increased in the *Rfwd2*^+/−^ animals during the probe test 24 h after training on day 6 (**J**) and 4 days after training on day 9 (**L**). The number of errors made before finding the target hole was increased on day 6 (**K**) but not on day 9 (**M**). The time spent in the target quadrant was decreased on day 6 (**N**) and day 9 (**O**) in *Rfwd2*^+/−^ mice compared to WT littermates. I Two-way repeated-measure ANOVA followed by Sidak’s test, others with two-population Student’s *t*-test. Data are presented as mean ± SEM. *p < 0.05; **p < 0.01. *n* = 10.

*Rfwd2*^flox/flox^ mice and *syn1-cre* mice showed normal behavior in the OFT, and similar spatial memory (SF. 1, 4). In the OFT, *Rfwd2*^+/−^ mice showed similar distance traveled as WT mice (Fig. 3A, B), the ratio of time spent in the center of the open field to the total distance traveled was significantly decreased in *Rfwd2*^+/−^ male mice (*p* < 0.05, Fig. 3C). *Rfwd2*^+/−^ mice also showed significant increases in the number of grooming events (*p* < 0.05, Fig. 3D) and the number of rearing events (*p* < 0.05, Fig. 3E) compared to their WT littermates. Nest building is an innate behavior that assesses activities of daily living [48]. *Rfwd2*^+/−^ male mice had significantly lower nesting task scores than WT littermates (*p* < 0.05, Fig. 3F, G). Taken together, *Rfwd2*^+/−^ male mice exhibited anxiety-like behaviors, increased repetitive behavior, and impaired nesting ability, all reminiscent of the symptoms of ASD [49].

### *Rfwd2*^+/−^ male mice show a deficit in spatial memory

In addition to the common abnormalities in communication and sociability, cognitive deficits are also common comorbidities of ASD [50]. Therefore, we performed Barnes maze tests to assess whether *Rfwd2*^+/−^ male mice have learning and memory deficits (Fig. 3H). After 1 day of habituation, mice were trained for 4 days to learn the location of the escape hole using spatial cues surrounding the platform. Acquisition training was followed by two probe trials 1 day and 4 days after training.

*Rfwd2*^+/−^ male mice showed a similar ability to learn the location of the escape hole during training as WT littermates (Fig. 3I). During the probe trials, *Rfwd2*^+/−^ male mice showed significant deficits in spatial memory as evidenced by an increase in escape latency in locating the target hole (Day 6, *p* < 0.05, Fig. 3J); (Day 9, *p* < 0.05, Fig. 3L), an increase in exploration errors (*p* < 0.01, Fig. 3K), and decreased time spent in the target quadrant (Fig. 3N, O), indicating spatial memory deficits.

### *Rfwd2*^+/−^ male mice show decreased spine density and synaptic protein levels and synaptic function deficits

**Fig. 4:**
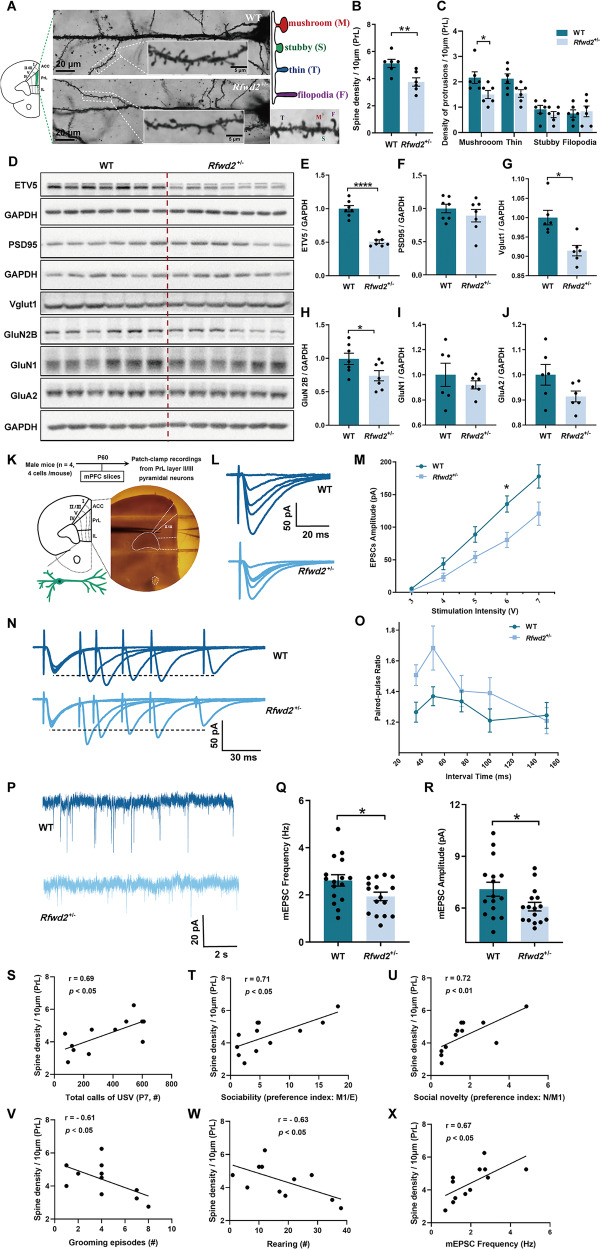
*Rfwd2*^+/−^ male mice showed decreases in both spine density and synaptic protein levels, as well as synaptic function deficits. **A** Golgi staining of dendritic spines of layer II/III pyramidal neurons in the prelimbic (PrL) area of the mPFC of 2-month-old WT and *Rfwd2*^+/−^ male mice; diagram and representative image show mushroom, stubby, and thin spines as well as filopodia. **B**, **C** There was a decrease in spine density and the number of mushroom spines on the PrL layer II/III pyramidal neurons in *Rfwd2*^+/−^ male mice compared to male WT littermates. **D**–**J** Western blot analysis showed significant decreases in Etv5, Vglut1, and GluN2B levels without significant changes in PSD95, GluN1, or GluA2 levels in the mPFC of *Rfwd2*^+/−^ male mice compared to WT male littermates. **K** Diagram showing whole-cell patch-clamp recording on the PrL layer II/III pyramidal neurons from acute mPFC slices. **L** Representative traces of evoked EPSCs from mPFC slices of male WT and *Rfwd2*^+/−^ mice. **M** Average peak amplitude of evoked EPSCs plotted as a function of stimulus intensity from WT and *Rfwd2*^+/−^ mice. **N** Representative traces of paired-pulse responses performed in mPFC slices. **O** Average peak paired-pulse ratio at interstimulus intervals of 35, 50, 75, 100, and 150 ms. **P** Representative traces of mEPSCs from mPFC slices of WT and *Rfwd2*^+/−^ mice. **Q**–**R** There were significant decreases in the frequency (**Q**) and amplitude (**R**) of mEPSCs. S-X. Pearson correlation analysis between spine density and total calls of USV (**S**), sociability (**T**), social novelty (**U**), grooming episodes (**V**), rearing numbers (**W**) and mEPSC frequency (**X**). **M**, **O** Two-way RM ANOVA followed by Sidak’s test, others with two-population Student’s t-test. Data are presented as mean ± SEM. *p < 0.05; **p < 0.01, ***p < 0.001. **A**–**J**, *n* = 6–7; **K**–**R**, *n* = 16 cells from 4 WT mice, *n* = 16 from 4 *Rfwd2*^+/−^ mice; **S**–**X**, *n* = 6.

The mPFC, particularly the PrL area plays a key role in anxiety, memory, and social behaviors [27–29], and dysfunction of the circuitry between PrL pyramidal neurons and downstream brain areas causes autistic-like behaviors [29, 30]. Since *RFWD2* is a high-risk gene for ASD [11], we sought to determine whether increased dosage of the *RFWD2* gene in *Rfwd2*^+/−^ mice would result in dysregulation of dendritic spines, a phenotype commonly observed in animal models of ASD and in patients with ASD [51, 52]. Indeed, we found that total spine density (thin, stubby, and mushroom) (*p* < 0.05, Fig. 4A, B) and mushroom spine number (*p* < 0.05, Fig. 4C) were significantly reduced on the apical dendrites of PrL layer II/III pyramidal neurons of *Rfwd2*^+/−^ male mice compared to those of WT littermates.

To determine whether RFWD2 affects the biochemical composition of synapses, we performed Western blot analysis and found that the levels of Vglut1 (*p* < 0.01, Fig. 4G) and NMDA receptor GluN2B (*p* < 0.05, Fig. 4H) proteins were significantly decreased in the mPFC of *Rfwd2*^+/−^ male mice compared to WT littermates, while no changes were detected in the levels of the synaptic protein PSD95, GluN1 or GluA2 (Fig. 4F, I, J). The levels of ETV5 protein, a major ubiquitination substrate of RFWD2, were significantly decreased in *Rfwd2*^+/−^ mice (*p* < 0.0001, Fig. 4E).

The PrL area plays an important role in the regulation of anxiety-like and social behaviors [29]. Given the observed alterations in spine density and synaptic proteins in *Rfwd2*^+/−^ male mice, we next examined synaptic function on PrL layer II/III pyramidal neurons in acute mPFC slices (Fig. 4K). We found that the average peak amplitudes of EPSCs were significantly different between WT and *Rfwd2*^+/−^ mice (interaction: *F*_4,71_ = 3.27, *p* < 0.05; stimulation intensity: *F*_1.76,31.23_ = 87.70, *p* < 0.001; genotype: *F*_1,18_ = 8.56, *p* < 0.01, Fig. 4M). The average peak amplitude of evoked EPSCs input/output was decreased in *Rfwd2*^+/−^ male mice (6 V: *p* < 0.05, Sidak’s test, Fig. 4L, M), indicating a decrease in excitatory synaptic strength in *Rfwd2*^+/−^ mice. The average peak paired-pulse ratios (PPRs) at interstimulus intervals of 35, 50, 75, 100, and 150 ms were analyzed, and *Rfwd2*^+/−^ male mice showed similar PPR (F_1,20_ = 2.20, *p* = 0.16) to WT littermates (Fig. 4N–O), indicating intact presynaptic function on PrL neurons.

We then examined spontaneous synaptic activities using mEPSC recordings on PrL layer II/III pyramidal neurons. Compared to WT controls, *Rfwd2*^+/−^ mice showed significant reductions in mEPSC frequency (*p* < 0.05) and amplitude (*p* < 0.05) (Fig. 4P–R). Interestingly, Pearson correlation analysis revealed a positive correlation between spine density and total USV calls (*r* = 0.69, *p* < 0.05, Fig. 4S). There were also strong positive correlations between spine density and sociability (*r* = 0.71, *p* < 0.05, Fig. 4T) and between spine density and social novelty (*r* = 0.72, *p* < 0.01, Fig. 4U). In contrast, negative correlations were observed between spine density and grooming episodes (*r* = −0.61, *p* < 0.05, Fig. 4V), and between spine density and rearing number (*r* = −0.63, *p* < 0.05, Fig. 4W). In addition, we found that spine density was positively correlated with mEPSC frequency (*r* = 0.67, *p* < 0.05, Fig. 4X), which is consistent with the decreased spine density and impaired synaptic proteins in *Rfwd2*^+/−^ male mice.

### *Rfwd2*^+/−^ female mice show mild deficits in social communication and spatial memory, as well as subtle deficits in synaptic structure and function

**Fig. 5:**
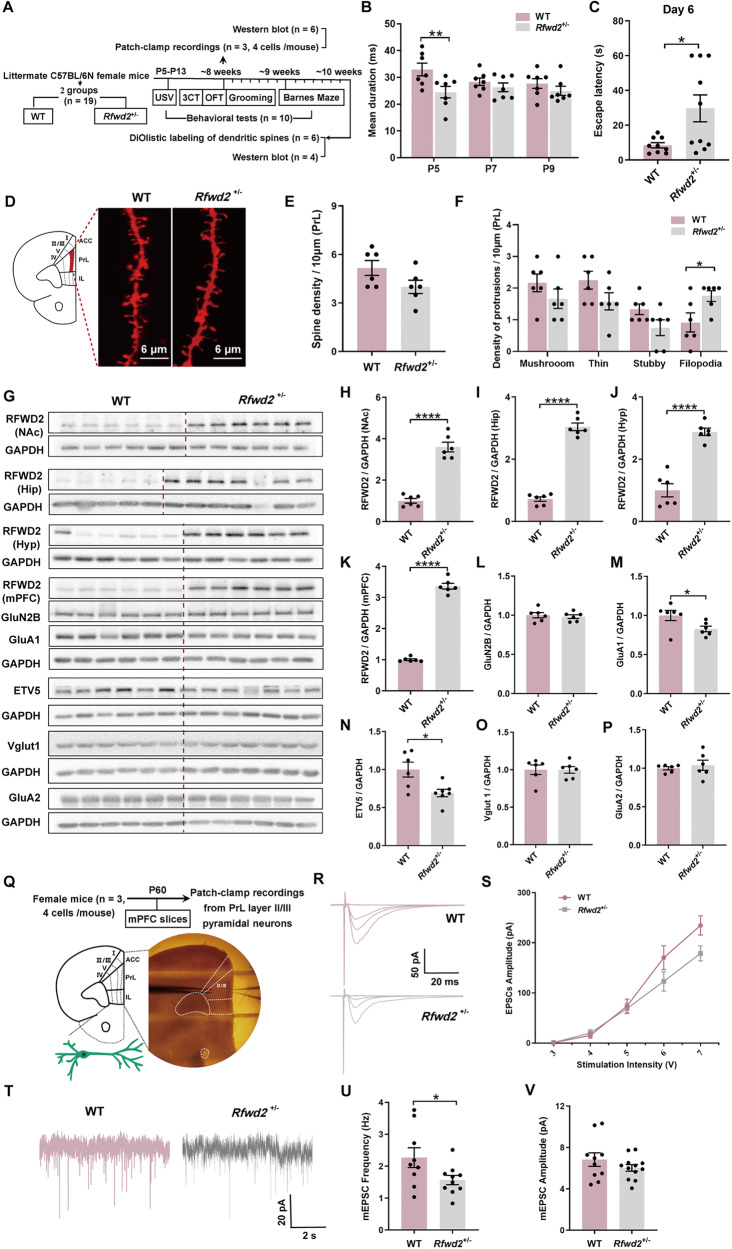
*Rfwd2*^+/−^ female mice showed normal spine density, abnormal morphology in spines, and decreased levels of related synaptic proteins, as well as a subtle deficit in synaptic function. **A** Experimental time course. **B** Mean call duration by *Rfwd2* ^+/−^ mice and WT female littermates on postnatal (P) days 5, 7, and 9. **C** Escape latency to the target hole on day 6 in the Barnes maze test. **D** Representative dendritic spines on the apical dendrites of layer II/III pyramidal neurons in the PrL of the mPFC labeled with Dil using a gene gun. **E**, **F** There was no change in total spine density, but an increase in the number of filopodia on PrL layer II/III pyramidal neurons in *Rfwd2*^+/−^ female mice compared to WT female littermates. **G**−**K** Analysis of RFWD2 protein by Western blot in the NAc, hippocampus (Hip), hypothalamus (Hyp) and mPFC of *Rfwd2*^+/−^ female mice and their wild-type (WT) littermates. **L**−**P** Western blot analysis showed a significant decrease in the levels of Etv5 and GluA1 without significant changes in GluN2B, Vglut1 or GluA2 in the mPFC of *Rfwd2*^+/−^ female mice compared to WT female littermates. **Q** Diagram showing whole-cell patch-clamp recording on the PrL layer II/III pyramidal neurons from acute mPFC slices. **R** Representative traces of evoked EPSCs from WT and *Rfwd2*^+/−^ female mice. **S** Average peak amplitude of evoked EPSCs plotted as a function of stimulus intensity from WT and *Rfwd2*^+/−^ female mice. Representative traces of mEPSCs from WT and *Rfwd2*^+/−^ female mice. **U** There was a significant decrease in frequency (**U**), and no change in amplitude (**V**) of mEPSCs. **B**, **S** Two-way RM ANOVA followed by Sidak’s test, others with two-population Student’s t-test. Data are presented as mean ± SEM. *p < 0.05; ****p < 0.0001. **A**–**P**, *n* = 6–7; **Q**–**V**, *n* = 9 cells from 3 WT mice, *n* = 12 from 3 *Rfwd2*^+/−^ mice.

With the identification of autistic-like characteristics in *Rfwd2*^+/−^ male mice, we wondered if female *Rfwd2*^+/−^ mice would have similar abnormalities. No differences in total body weight gain were observed between WT and *Rfwd2* ^+/−^ female mice during development (SF. 5A). Data from the isolation-induced USV test were analyzed by two-way RM ANOVA followed by Sidak’s test, and showed that the total five-min call durations in *Rfwd2*^+/−^ female pups at P5, P7, and P9 were comparable to those of WT littermates (SF. 5D). A decrease in the mean duration of calls in *Rfwd2*^+/−^ females was found only at P5 (*p* < 0.01), but not at P7 or P9 (Fig. 5B). There were no significant differences in the total time spent in calls at P5, P7 or P9 between the two groups (SF. 5B). In the OFT, *Rfwd2* ^+/−^ females showed no differences from WT littermates in distance traveled (SF. 5E) or in the ratio of time spent in the center to time of total distance traveled (SF. 5F), and both groups showed similar numbers of grooming events (SF. 5G, H). In the three-chamber social test, both *Rfwd2*^+/−^ and WT female mice spent more time with the novel mouse 1 (M1) than with the empty (E) chamber in sociability sessions (SF. 5I, J), and more time with the novel mouse N than with familiar mouse M1 in social novelty sessions (SF. 5L, M). There were no differences between *Rfwd2*^+/−^ females and WT littermates in either the preference index (M1/E) in the sociability test (SF. 5K) or the preference index (N/M1) in the social novelty test (SF. 5N). In the Barnes maze test (SF. 5O), *Rfwd2*^+/−^ females were able to locate the escape hole during training sessions at the levels similar to WT females (SF. 5P). However, during the probe trials, *Rfwd2*^+/−^ females showed a deficit in spatial memory as evidenced by an increase in escape latency to locate the target hole (*p* < 0.05) on day 6 (Fig. 5C), but not on day 9 (SF. 5R). Compared to WT, *Rfwd2*^+/−^ females did not show a significant difference in the number of errors made in locating the target hole on days 6 or 9 (SF. 5S, T), or in the time spent in the different quadrants (SF. 5U, V). These results indicate that *Rfwd2*^+/−^ *f*emale mice showed subtle deficits in social communication and spatial memory, but were not significantly affected in anxiety-like, repetitive, or social behaviors.

To examine neuronal structural development, dendritic spines on PrL layer II/II pyramidal neurons were labeled by delivering lipophilic dye-coated particles to mPFC slices, and spines were classified into thin, stubby, and mushroom subtypes. Filopodia are considered the precursors of dendritic spines and were not included in spine density analysis. Spine densities were similar between the two groups, whereas filopodia were increased in *Rfwd2*^+/−^ females compared to WT littermates (*p* < 0.05, Fig. 5D–F). The estrous cycle is not determined in female mice. Previous studies suggest that spine density on the mPFC pyramidal neurons is stable throughout the estrous cycle in female mice [37–41]. However, we cannot completely exclude the possible role of the estrous cycle in spine density changes on PrL layer II/III pyramidal neurons. As expected, RFWD2 expression in *Rfwd2*^+/−^ females was increased in brain regions including the nucleus accumbens (NAc) (*p* < 0.0001, Fig. 5H), hippocampus (*p* < 0.0001, Fig. 5I), hypothalamus (*p* < 0.0001, Fig. 5J), and mPFC (*p* < 0.0001, Fig. 5K) compared to WT littermates. Regarding the biochemical composition of synapses, GluA1 levels (*p* < 0.05, Fig. 5M) were significantly decreased in *Rfwd2*^+/−^ females, while no changes were detected in the levels of GluN2B, Vglut1 or GluA2 (Fig. 5L, O, P). ETV5 protein levels were significantly decreased in *Rfwd2*^+/−^ females (*p* = 0.01, Fig. 5N). For the synaptic function in the PrL area (Fig. 5Q), no significant changes were detected in the average peak amplitude of evoked EPSCs input/output (Fig. 5R, S). Recordings of 3-minute mEPSCs showed a reduction in mEPSC frequency (*p* < 0.05, Fig. 5T, U), but not in amplitude (Fig. 5V). These results indicate an impairment of excitatory synapse function on PrL neurons, consistent with the abnormal spine morphology and altered GluA1 levels in *Rfwd2*^+/−^ female mice.

### Restoring ETV5 in the mPFC rescues impaired sociability in *Rfwd2*^+/−^ male mice

**Fig. 6:**
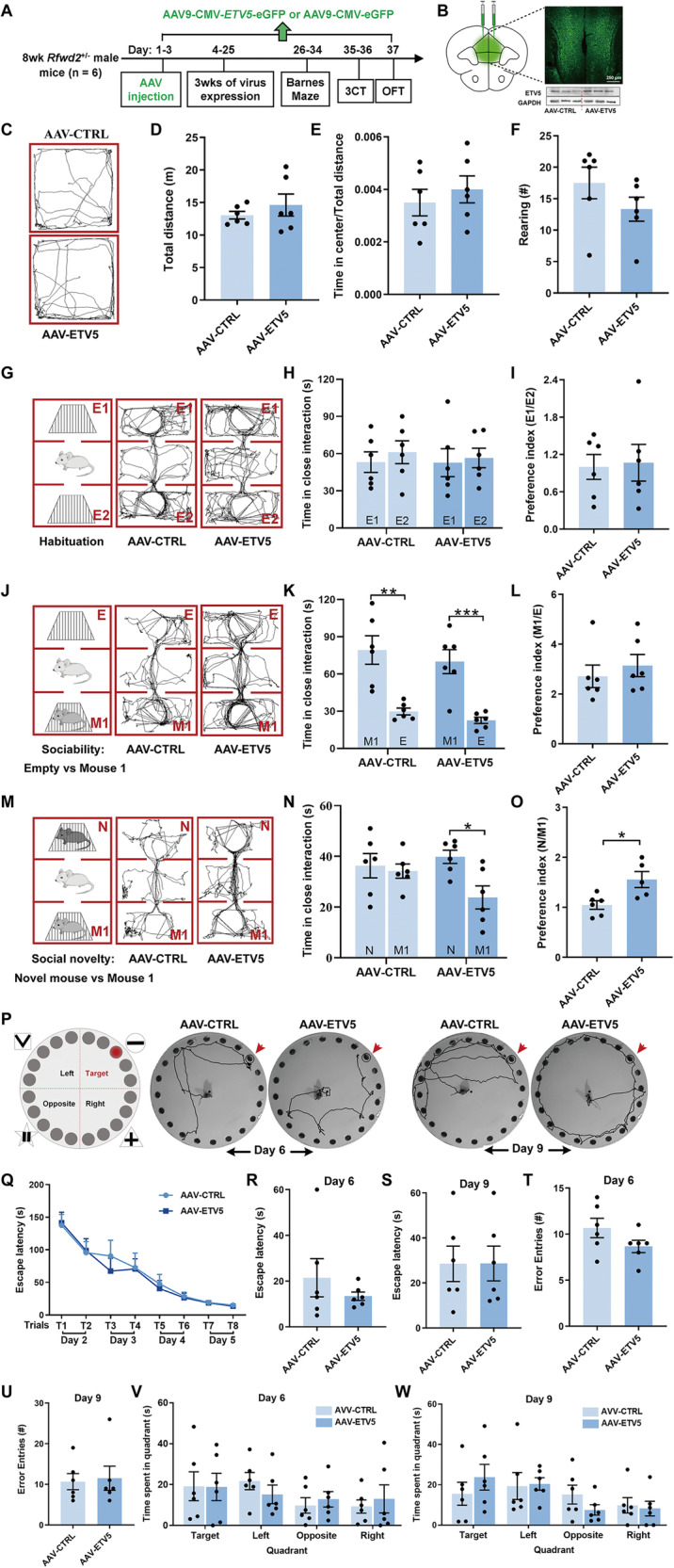
Restoration of ETV5 in the mPFC rescues impaired sociability in *Rfwd2*^+/−^ male mice. **A** Experimental design involving injection of the AAV9-CMV-*ETV5*-eGFP encoding ETV5-eGFP (AAV-ETV5) or empty vector AAV9-CMV-eGFP encoding EGFP only [AAV-CTRL (control), empty vector control without ETV5] into the bilateral mPFC of *Rfwd2*^+/−^ mice (*n* = 6, 60 days old); behavioral tests were conducted 3 weeks post-injection. 3CT: 3-chamber test. OFT: open field test. **B** Representative confocal image showing the expression of AAV-CMV-*ETV5*-eGFP in the mPFC. **C** Representative traces of an ETV5-expressing or vector control *Rfwd2*^+/−^ mouse in the OFT. **D**–**F** The ETV5-expressing mice showed no alterations in locomotion (**D**), time spent in the center (**E**), or rearing (**F**) in the OFT compared to controls. **G**–**O** Expression of ETV5 rescued impaired social behavior in the 3-chamber test in *Rfwd2*^+/−^ male mice. **G** Habituation to the three-chamber apparatus. ETV5-expressing and vector control mice showed similar amounts of time spent in the empty cages (**H**) and preference index for two empty cages (**I**). **J** Schematic of the sociability test. ETV5-expressing and vector control *Rfwd2*^+/−^ mice showed similar interaction times with mouse 1 (**K**) and preference index (**L**) for an unfamiliar mouse 1 (M1), but ETV5-expressing mice showed increased interaction time with a novel mouse (**N**) and increased preference index (**O**) compared to control mice. **M** Representative traces from an ETV5-expressing or vector control mouse in the ‘Familiar Mouse1–novel mouse’ test. **P**–**W** Expression of ETV5 did not rescue the spatial memory deficit in *Rfwd2*^+/−^ mice. **P** Experimental diagram of the Barnes maze, and representative traces of the probe trials on days 6 and 9. **Q** Escape latency during training trials on days 2–5. Latency to locate the escape hole (**R**, **S**), error entries to locate the target (**T**, **U**), and time spent in the target quadrant (**V**, **W**) 24 h and 4 days after training on days 6 and 9 were not different between ETV5-expressing and control mice, respectively. **H**, **K**, **N** two-way ANOVA followed by Bonferroni’s test. **Q** Two-way repeated-measures ANOVA followed by Sidak’s test, others with two-population Student’s *t*-test. Data are presented as mean ± SEM. *p < 0.05; **p < 0.01, ***p < 0.001.

As one of the major substrates of RFWD2, ETV5 has been implicated in emotion modulation, and spine development [26]. A reduction in Etv5 protein levels (Fig. 4E) was accompanied by autistic-like, anxiety-like behaviors, and a deficit in spatial memory in *Rfwd2*^+/−^ male mice. To investigate whether alterations in ETV5 contribute to the expression of these behavioral phenotypes, we injected AAV9-*ETV5*-eGFP (AAV-ETV5) or empty control AAV9-eGFP (AAV-CTRL) into the bilateral mPFC of *Rfwd2*^+/−^ male mice at P60 (Fig. 6A, B), and behavioral tests were performed 3 weeks after virus injection. We found that ETV5 expression did not have a significant effect on the total distance traveled, the time spent in the center, or the number of both grooming and rearing events in the open field (Fig. 6C–F). In the habituation session of the 3-chamber test, ETV5-expressing *Rfwd2*^+/−^ mice and control mice expressing eGFP spent similar amounts of time in empty (E)1 and E2 cages and showed similar preferences for E1 and E2 cages (Fig. 6G–I). These two groups of mice also showed similar interactions with the novel mouse M1 and had similar preferences index for the novel mouse M1 (Fig. 6J–L). In the social novelty session, two-way ANOVA showed a significant effect of ETV5 on interaction time (F_1,20_ = 5.64, *p* < 0.05, Fig. 6N). Bonferroni’s test showed that ETV5-expressing *Rfwd2*^+/−^ mice spent more time interacting with the novel mouse N than with the familiar mouse M1 (*p* < 0.05, Fig. 6M, N), and showed a significant increase in the preference index compared to control mice (*p* < 0.05, Fig. 6O). These results indicate that restoration of ETV5 was able to rescue the social deficits in *Rfwd2*^+/−^ male mice. Two-way RM ANVOA showed that that ETV5-expressing *Rfwd2*^+/−^ male mice showed no change in escape latency during the training trials (F_1,10_ = 0.12, *p* = 0.73, Fig. 6Q) in the Barnes maze test. There were no differences in the latency to locate the target hole (Fig. 6R, S), error entries to locate the target hole (Fig. 6T, U), the time spent in the target quadrant (Fig. 6V, W) between ETV5-expressing and control mice on days 6 and 9, respectively, during the probe trail. Thus, expression of ETV5 in the mPFC was unable to rescue the spatial memory deficits. To determine the specificity of this Etv5 rescue in *Rfwd2*^+/−^ males, we examined whether expression of ETV5 in the mPFC of WT male littermates resulted in behavioral changes. We injected the WT-ETV5 and WT-CTRL viruses, respectively, into the bilateral mPFC of WT male mice as described in SF. 6A. ETV5 protein levels were significantly increased in the WT-ETV5 group compared to WT-CTRL mice (*p* < 0.05, SF. 6B). ETV5 expression had little effect on the total distance traveled, the time spent in the center, grooming number, or rearing number in the OFT (SF. 6C–F). WT-ETV5 mice and WT-CTRL mice showed similar social preferences (SF. 6G–O), and similar spatial learning and memory abilities (SF. 6P–W). Thus, expression of ETV5 in the mPFC did not alter social behavior in WT male mice.

## Discussion

Genome-wide data have shown an association of *RFWD2* gene copy number variations with autism manifested as the presence of 3 copies of *RFWD2* in certain ASD patients [11]. We have generated a conditional *RFWD2* knockin *Rfwd2*^+/−^ mouse model of autism that contains an extra copy of *RFWD2* to mimic the 3-copy *RFWD2* ASD condition. As expected, *Rfwd2*^+/−^ mice showed an increased in RFWD2 protein expression in different brain regions and showed normal body weight and gross brain morphology during postnatal development compared to WT littermates. *Rfwd2*^+/−^ male mice showed typical autistic-like, anxiety-like behaviors, and spatial memory deficits, while *Rfwd2*^+/−^ female mice showed only subtle deficits in communication and spatial memory.

### *Rfwd2*^+/−^ male, but not female mice, showed autistic-like behaviors

Social interaction deficits, impaired communication, and repetitive behaviors are core symptoms of ASDs [53]. *Rfwd2*^+/−^ male mice exhibited a significant impairment in communication characterized by the increased use of simpler and shorter call types, fewer calls, and less time spent in making calls compared to their WT male littermates, consistent with other mouse models of ASDs [54]. *Rfwd2*^+/−^ male mice also showed significant impairments in interacting with novel mice, decreased sociability, and loss of interest in social novelty. The behavioral impairments in *Rfwd2*^+/−^ male mice recapitulates the impaired social behavior in patients with ASD, supporting the genome-wide data [11]. Repetitive behavior was evaluated by grooming [55]. *Rfwd2*^+/−^ male mice showed a significant increase in grooming, similar to other mouse models of ASD [14].

ASD has a strong gender bias with a male to female prevalence ratio of approximately 4:1 [5]. Indeed, *Rfwd2*^+/−^ female mice showed only subtle impairments in social communication, characterized by a shorter duration of USV calls at P5, but not at P7 or P9, indicating normal sociability and social novelty. In contrast, *Rfwd2*^+/−^ male mice showed a greater deficit in social communication. The ability of females to hide their autistic traits by reducing their social hurdles is a “camouflaging”-type behavior [56, 57]. The “camouflaging”-type behavior was also observed in juvenile neurofibromatosis type 1 mutant mice [4]. The lack of autistic-like behaviors in *Rfwd2*^+/−^ female mice may result from an innate protective factor in females [58], that reduces the expression of autistic-like behaviors when the genetic risk for autism is equivalent to that of males [57]. The sex-specific neurobehavioral profile of *Rfwd2*^+/−^ mice shows similarities to the different clinical phenotypes observed in male and female autistic patients [5, 58]. Thus, *Rfwd2*^+/−^ mice could serve as a model to study the mechanisms underlying “camouflaging”-type behavior [56, 57]. Male and female sex hormones may contribute to the sex differences in autistic-like behaviors in *Rfwd2*^+/−^ male mice, which will be a direction for future studies.

### *Rfwd2*^+/−^ male, but not female mice, exhibited anxiety-like behaviors and impaired nest building

Anxiety is often comorbid with ASDs [1]. Mice with increased levels of anxiety-like behavior prefer to spend less time in the center of the open field in the OFT [14, 29]. *Rfwd2*^+/−^ male, but not female, mice spent less time in the center of the open field compared to WT littermates, consistent with other mouse models of ASD [14, 29].

Nesting behavior is an innate activity of daily life in many animals and correlates with social behavior in mouse models of ASD [32]. *Rfwd2*^+/−^ male mice had difficulty in building a nest from the provided nesting materials, showing impaired nesting behavior that is also found in other mouse models of ASD, such as *Nlgn4* null mutant mice [14, 59]. Impaired nesting behavior may be associated with ASD individuals with varying degrees of impairment in daily living skills [60].

### *Rfwd2*^+/−^ male mice showed decreased spine density, synaptic function deficits, and impaired spatial memory

Alterations in spine density, synapse number, and synaptic function are implicated in ASD [61]. *Rfwd2*^+/−^ male, but not female, mice showed a decrease in spine density on the PrL layer II/III pyramidal neurons as seen in other mouse models of ASD [62]. Dendritic spines of PrL pyramidal neurons receive extrinsic glutamatergic inputs from other brain areas and provide the structural basis for behaviors [63, 64].

*Rfwd2*^+/−^ male mice showed spatial memory deficits that are consistent with impaired spatial memory in other mouse models of ASD and memory deficits in some patients with ASD [14]. Based upon normal locomotion in the OFT, the errors in finding the escape hole in the spatial cued-recall task were not likely due to alterations in processing the visual cues [65] or motor ability, but were likely due to deficits in brain circuitry as suggested by a positive correlation between poor spatial memory and decreased spine density on PrL neurons in *Rfwd2*^+/−^ male mice. The synaptic connections between PrL layer II/III pyramidal neurons and other brain areas [66, 67] are important for social behavior, communication, cognition and anxiety [30, 45]. Dysregulation of these PrL projections causes autistic-like and anxiety-like behaviors [29]. Decreased spine numbers in *Rfwd2*^+/−^ male mice is a key component of the neuropathological events underlying ASD.

The dendritic spine is highly dynamic, and spine density and morphology correlate with synaptic function [68]. There is a positive correlation between spine size and synaptic strength; a mushroom spine has better synaptic efficacy than a thin spine [69]. The head of the mushroom spine is stable, contains a high postsynaptic density and carries a high concentration of glutamate receptors, scaffolding molecules, and other proteins essential for postsynaptic function. A decrease in spine density, particularly on the mushroom spine, was accompanied by a corresponding decrease in the input/output response as well as the frequency and amplitude of mEPSCs, indicating an impairment in excitatory synaptic transmission in *Rfwd2*^+/−^ male mice, which is consistent with previous findings in other mouse models of ASD [30, 70–72]. Decreased spine density on the PrL layer II/III pyramidal neurons was accompanied by decreased levels of both Vglut1 and GluN2B protein in *Rfwd2*^+/−^ male mice. Vglut1 plays a critical role in excitatory signaling [73]. A decrease in synaptic proteins is associated with autistic-like behaviors [17]. Normal GluN2B function is essential for normal dendritic spines, synaptic function, and healthy behavior [74–76]. Spine dynamics are both a cause and a consequence of behavior [63], and spine density is correlated with behavior [77, 78]. Thus, an increase in *Rfwd2* gene dosage leads to impairments in synapse formation and function, which likely results in disruption of neural circuit function that leads to the expression of autistic phenotypes [79, 80].

### Restoration of ETV5 in the mPFC of *Rfwd2*^+/−^ male mice rescued deficits in social behavior

The mPFC dysfunction contributes to autistic-like behaviors [29, 30]. As one of the key substrates of RFWD2, ETV5 plays a role in spine formation, spatial memory, and social behavior [26]. We showed that restoring ETV5 expression in the mPFC of *Rfwd2*^+/−^ mice rescued the impaired social behavior of *Rfwd2*^+/−^ male mice, demonstrating a key role of ETV5 [26]. In contrast, restoration of ETV5 in the mPFC did not rescue the spatial memory deficits or the anxiety-like behaviors. The lack of effect on memory is consistent with previous findings that the mPFC does not play a key role in spatial memory [81]. ETV*5* mRNA is predominantly expressed in the cortex, including the mPFC, amygdala, and hypothalamus [82]. It is likely that decreased Etv5 expression in other brain regions contributes to anxiety-like behavior in *Rfwd2*^+/−^ male mice, so that expression of ETV5 in the mPFC is probably not sufficient to rescue the anxiety deficits. We also showed that overexpression of ETV5 in the mPFC of WT mice had no effect on social behavior, indicating that the rescue effect of ETV5 virus injection in *Rfwd2*^+/−^ male mice is not due to additional independent effects of elevated ETV5, but is rather a consequence of normalizing the reduced ETV5 levels in *Rfwd2*^+/−^ mice.

### The RFWD2-dependent ubiquitin-proteasome pathway contributes to autistic-like behaviors in *Rfwd2*^+/−^ male mice

Proteolysis of specific substrates by the UPS regulates several important neural processes that are impaired in ASD [22, 83]. The UPS is also involved in both the pre- and postsynaptic compartments, regulating synaptic properties and dynamic postsynaptic changes on dendritic spines [83]. RFWD2-mediated changes on dendritic spines and synaptic plasticity may be regulated by the levels of its substrates such as ETV5 via ubiquitination, in accordance with ETV5’s function in spine formation [26]. Deficiency of the E3 ligase *Cullin 3* in the forebrain has been shown to induce degradation of NMDA receptors, leading to synaptic function deficits and autistic-like behaviors [84]. Etv5 protein levels are significantly decreased in the mPFC of *Rfwd2*^+/−^ male mice, presumably due to RFWD2-dependent increased protein ubiquitination and degradation.

In conclusion, *Rfwd2*^+/−^ male mice exhibited autistic-like behaviors, which were accompanied by decreased spine density, reduced levels of both Etv5 and synaptic proteins, and synaptic function deficits. Restoration of ETV5 in the mPFC rescued the impaired social behavior. Our findings reveal an important role for RFWD*2* in synapse formation, function, and ASD pathogenesis, and provide novel insights into the mechanism underlying RFWD2-related ASD.

## Supplementary information

Supplementary Methods

Supplementary Figures

Supplementary Table 1

## Data Availability

All data is available upon reasonable request.
