# Correction: Increased gene dosage of *RFWD2* causes autistic-like behaviors and aberrant synaptic formation and function in mice

**DOI:** 10.1038/s41380-024-02634-1

**Published:** 2024-07-16

**Authors:** Yong-Xia Li, Zhi-Nei Tan, Xu-Hui Li, Boyu Ma, Frank Adu Nti, Xiao-Qiang Lv, Zhen-Jun Tian, Riqiang Yan, Heng-Ye Man, Xin-Ming Ma

**Affiliations:** 1 College of Life Sciences, Shaanxi Normal University, Xi’an, China; 2 Center for Neuron and Disease, Frontier Institutes of Science and Technology, Xi’an Jiaotong University, Xi’an, China; 3 Department of Oral and Maxillofacial Surgery, University of Alabama at Birmingham, Birmingham, AL USA; 4 Institute of Sports Biology, College of Physical Education, Shaanxi Normal University, Xi’an, China; 5 Department of Neuroscience, University of Connecticut Health, Farmington, CT USA; 6 Department of Biology, Boston University, Boston, MA USA

**Keywords:** Autism spectrum disorders, Psychology

Correction to: *Molecular Psychiatry* 10.1038/s41380-024-02515-7, published online 19 March 2024

In this article the statement in the Funding information section was incorrectly given as ‘This work was supported by a research fund from Shanxi Normal University, the Fundamental Research Funds for the Central Universities to YXL (2018TS075), the National Science Foundation of China (32100810) and the China Postdoctoral Science Foundation (2022M710111) to XHL’ and should have read ‘This work was supported by a research fund from Shaanxi Normal University, the Fundamental Research Funds for the Central Universities to YXL (2018TS075), the National Science Foundation of China (32100810) and the China Postdoctoral Science Foundation (2022M710111) to XHL’.

The original article has been corrected.

