# Supplementary material for: Increased gene dosage of RFWD2 causes autistic-like behaviors and aberrant synaptic formation and function in mice
Source: Mol Psychiatry. 2024 Mar 19;29(8):2496–509. doi: 10.1038/s41380-024-02515-7 (PMC11412905; doi:10.1038/s41380-024-02515-7)
Supplement: Supplementary file 1 — Supplementary Methods [file 41380_2024_2515_MOESM1_ESM.docx]

**Increased gene dosage of *RFWD2* causes autistic-like behaviors and aberrant synaptic formation and function in mice**

Yong-Xia Li^1^, Zhi-Nei Tan^1^, Xu-Hui Li^2^, Boyu Ma^3^, Frank Adu Nti^1^, Xiao-Qiang Lv^1^, Zhen-Jun Tian^4^, Riqiang Yan^5^, Heng-Ye Man^6^*, Xin-Ming Ma^5^*

^1^College of Life Sciences, Shaanxi Normal University, Xi’an, China

^2^Center for Neuron and Disease, Frontier Institutes of Science and Technology, Xi’an Jiaotong University, Xi’an, China

^3^Department of Oral and Maxillofacial Surgery, University of Alabama at Birmingham, Birmingham, AL, United States

^4^Institute of Sports Biology, College of Physical Education, Shaanxi Normal University, Xi'an, China

^5^Department of Neuroscience, University of Connecticut Health, Farmington, CT, United States

^6^Department of Biology, Boston University, Boston, Massachusetts, United States

**Supplementary methods**

**Generation of conditional *Rfwd2* knockin mutant mice**

CRISPR/Cas9-based extreme genome editing (EGE) technique was used to generate *Rfwd2* conditional knock-in mice in the C57BL/6N background (Biocytogen, Beijing). The mouse *Rfwd2* gene (gene ID: 26374), including 13 transcripts, spans about 123kb on the forward strand of chromosome 1. The Rosa26 locus is one of the ideal sites where a gene of interest can be targeted in the mouse. Therefore, the targeting vector “CAG-Pr-LoxP-Stop-LoxP-*Rfwd*2-WPRE-pA” was inserted into the intron between exons 1 and 2 of the ROSA26 locus (Fig. 1A). The LoxP-Stop-LoxP cassette was used to control Cre-mediated conditional activation of the Rfwd2 gene. To screen for the best sgRNA, a total of 18 single guide RNAs (sgRNAs) were designed and cloned into a pCS-3G vector encoding CAS9 by the Gibson assembly method. sgRNA12, which showed the highest CAS9/sgRNA activity, was chosen and cloned into a plasmid vector with a T7 promoter, which was then transcribed in vitro to obtain RNA for microinjection. Cas9/sgRNA and the targeting vector were microinjected into mouse zygotes isolated from C57BL/6N mice. The genotype of F0 mice after injection was confirmed by PCR. Positive F0 mice were mated with wild-type C57BL/6N mice to produce F1 generation mice, which were identified by PCR. Southern blot and sequencing were used to ensure the correct recombination without random insertion in F1 mice. The F1 mice were backcrossed onto C57BL/6N mice for two generations to generate Rfwd2 conditional knockin-floxed mice (Fig. 1A).

Synapsin1 (Syn1)-Cre mice that were developed on the *C57BL/6N background* were backcrossed onto C57BL/6N mice for two generations prior to any experiments. *Rfwd2* knockin-floxed homozygous mice were crossed with Syn1-Cre mice to generate heterozygous Rfwd2 knockin mutant (*Rfwd2^+/-^)* mice expressing 3 copies of RFWD2. Heterozygous *Rfwd2^+/-^* mice were then intercrossed to generate homozygous Rfwd2 mutant (*Rfwd2^+/+^*) mice expressing 4 copies of RFWD2, together with additional heterozygous *Rfwd2^+/-^* mice and wild-type (WT) littermates. *Rfwd2^+/-^* male and female mice and their WT littermates of both sexes were used in this study.

**Open field test (OFT)**.

The OFT was performed as described previously^1^. Briefly, the box was a 50cm × 50cm × 35cm container, evenly divided into 9 grids. The mice were briefly handled each day for 5 consecutive days prior to testing. On the day of the test, the mice were allowed to habituate to the test room for 1 h before the start of the test. A single mouse was placed in the center of the open field, and its activity was recorded for 5 min with Video Mot 2 (TSE, German). Between tests, the box was wiped with 70% alcohol. Total distance traveled, rearing, grooming, and the time spent in the central area of the open field were analyzed by an experimenter blinded to treatment conditions.

**Ultrasonic vocalization (USV) recording**

On postnatal days 5, 7 and 9 (P5, P7, P9), one pup from each litter of the WT and *Rfwd2*^+/-^ groups (10 litters in total) was selected for the 5-minute USV recording session using a microphone. Pups were gently placed in a sound-attenuating box (21 cm × 21 cm × 21 cm) containing fresh, clean bedding. Vocalizations were recorded at 250 kHz in 16-bit format. The stored sound files were analyzed using BatSound 4.2 after a fast Fourier transform (512 FFT length, 100% frame, Hamming window, and 75% time window overlap), and a high-pass filter was applied to eliminate background noise < 30 kHz ^2^. The number and mean/total duration of each ultrasonic call were measured.

**Three chamber social test**

The box was divided into three chambers (center and two side chambers) by two partitions with 4 cm × 7 cm cutout doors allowing access to each chamber. The two side chambers contained small empty wire cages with many slits to allow direct nose-to nose/tail social contact between the mice. The test consisted of three sessions: habituation, sociability, and social novelty preference ^3^. The first session began with a 10-min habituation session in which the test mouse was allowed to explore all the three chambers. The habituation session was followed by a sociability test session in which a sex- and weight-matched novelty mouse was placed in one of the small wire cages in one of the side chambers as mouse 1 (M1), and the other small cage served as a non-social stimulus. The final session was the social novelty test, where another unfamiliar mouse was placed in the small cage in the other side chamber as the novel mouse (N). Before the start of each session, the test mouse was placed in the center chamber, and allowed to explore freely for 10 min. Video recordings were made using a Logitech webcam. Time spent interacting with each mouse or empty cage (nose ≤ 2 cm) and locomotion tracks were scored using the Smart 3.0 system.

**Nesting behavior**

Mice were individually housed in a new home cage without environmental enrichment for 3 days of acclimation, and on the fourth day 3.0g compressed cotton squares were placed in each cage as nesting material at 6:00 pm, and the nests were scored 14 hours later. Nest scoring was performed by observers blinded to animal genotype using a 5-point rating scale^4^.

**Barnes maze test (BMT)**

The Barnes maze setup consists of an elevated circular white platform (0.92 m in diameter) with twenty holes around the perimeter of the maze. The escape box, which consists of a black metal box, is mounted under one of the holes, while the remaining 19 holes are empty. The open, lit environment served as an aversive factor motivating the mice to find the hole with the escape box. The platform and the escape box were thoroughly cleaned with 70% ethanol. The test consisted of three sessions: habituation, acquisition trial, and probe trial. The habituation trial was conducted on the first day only. After being placed in the escape box for 1 min, the animal was placed in the center of the maze covered with an opaque cardboard chamber for 15 s, and then given 5 min to locate and enter the escape box. An animal that failed to locate the escape box was directed into it and remained there for 1min. The acquisition trials lasted for 4 consecutive days (days 2-5, 2 times/mouse/day, 8 times in total). The escape box was fixed in a new location that was different from the one used during habituation. During this trial, the mice were given 3 min to locate and enter the escape box and if they failed, they were gently guided into the box. On days 6 and 9, a 1-min probe trial was performed without the escape box. Escape latency, incorrect entries into the target tunnel, and time spent in four different quadrants were recorded. At the end of the behavioral studies, brain tissue was harvested for further studies such as dendritic spine analysis.

**Golgi staining**

Mice were anesthetized with isoflurane and were fixed by perfusion with 4% paraformaldehyde (PFA). Whole brains were immersed in Golgi-Cox solution for 14 days in the dark, and then were transferred to 30% sucrose solution until the brains sank to the bottom of the tube. The brains were sectioned at 100 µm thickness using a vibratome. Sections were collected on gelatin-coated slides for further processing. After drying, sections were processed sequentially through dH_2_O (1 min), 28% ammonium hydroxide (40 min in the dark), dH_2_O (1 min), acidic hardening solution (40 min, in the dark) and dH_2_O (2 × 1 min). After dehydration and clearing in graded alcohol solutions and xylene, the sections were coverslipped. The mPFC PrL was identified based upon its anatomical localization (**Fig. 5A**)^5^ and by comparing the landmarks with standard maps of the mouse brain. Layer II/III pyramidal neurons were identified based upon their location and morphology, characterized by large size and a well-impregnated dendritic tree, and by comparison with the adjacent Nissan-stained section. Z-step images were acquired with a light microscope (Axio Observer.Z1 with ApoTome2, Zeiss, Germany), and the optimal Z-step was recommended by the software (ZEN 2.3, Zeiss, Germany). The second-order segment on the apical dendrite of layer II/III pyramidal neurons in the PrL area of the mPFC was analyzed as described in the dendritic spine analysis below.

**DiOlistic labeling of dendritic spines**

Mice were perfusion-fixed with 4% PFA followed by 1-hour post-fixation in 4% PFA after mice were completely anesthetized with isoflurane, which produced the clearest images with the most readily visible spines^6^. DiI labels cell membranes and provides a better visualization of dendritic spines than Golgi staining. Sections (100μm) containing the mPFC were prepared using a vibratome and were then diolistically labeled using a Gene Gun (*Bio-Rad*, Hercules CA). A stack of images (Z-step, 0.2 μm with 63x objective) of the apical dendrites on layer II/III pyramidal neurons of the PrL area was captured under the same conditions^6^ using a Zeiss LSM 800 confocal microscope by an experimenter blinded to treatment conditions.

**Dendritic spine analysis**

Spine density was counted after images were calibrated and thresholds were set to ensure that all structures of interest were included in the analysis, and when spines were no longer than 3 μm in length ^7, 8^. Spines were categorized into three types: thin, mushroom, and stubby^5^. Spines were considered to be thin when the length was greater than the neck diameter, and the diameters of the head and neck were similar. Spines were considered to be mushroom when the diameter of the head was much larger than the diameter of the neck. Spines were considered to be stubby if the length and width were equal. Dendritic filopodia were defined and counted if length was >3 µm and <10 µm ^7, 8^, but filopodia were not included in spine density.

**Western blot**

Brain samples from *Rfwd2*^+/-^ mice and their WT littermates were dissected on ice. Coronal sections were cut using an ice-cold metal mouse brain matrix (0.5mm, RWD Life Science). Bilateral hippocampus and hypothalamus were dissected with micro forceps. Bilateral mPFC and NAc tissues were obtained using a 1mm diameter stainless steel stylet and stored at -80℃ until processed. Protein concentrations were determined using a BCA protein assay kit. Protein samples were separated by SDS-PAGE, transferred to PVDF membranes, and blocked in 5% milk for 2 hours at room temperature. The membranes were then probed with RFWD2 (Sigma, # WH0064326M1, 1:1000), ETV5 (Thermo Fisher Scientific, # PA5-30023, 1:1000), PSD95 (Cell Signaling Technology, # 3450, 1:1000), Vgult1 (Merck, # AB5905, 1:1000), GluN2B (Cell Signaling Technology, # 4207, 1:1000), GluN1 (Cell Signaling Technology, # 5704, 1:1000), GluA2 (Cell Signaling Technology, # 13607, 1:1000), GAPDH (Beijing Zhong Shan - Golden Bridge Biological Technology, # TA-08, 1:10000) overnight at 4°C followed by horseradish peroxidase-conjugated secondary antibodies for 1 hour at room temperature. Signals were acquired using Tanon Fine DoX6+Multi software. Quantification was performed using Tanon GIS software. The results were normalized to the corresponding GAPDH.

**Nissl staining and cell count analysis**

Nissl staining with crystal violet was performed on 12 µm cryotome coronal sections to check morphology and to assess cell number. The sections were then dehydrated through graded alcohol solutions, cleared in xylene, and mounted on slides with neutral balsam. Brain sections were visualized under a light microscope using an EVOS FL Auto 2 system (Thermo Fisher). Exposure time was kept constant throughout the acquisition of all images. Neurons were counted using NIH Image J software, and the results are presented as number of neurons per mm^2^.

**Electrophysiological recordings on mPFC brain slices.**

Eight-week-old mice were anesthetized with isoflurane, and the brains were rapidly removed and placed in ice-cold, oxygenated (95% O_2_ and 5% CO_2_), high-sucrose cutting solution containing: 252 mM sucrose, 25 mM NaHCO_3_, 10 mM glucose, 2.5 mM KCl, 1.2 mM NaH_2_PO_4_, 6 mM MgSO_4_, and 0.5 mM CaCl_2._ Acute coronal brain slices containing the mPFC (300 μm) were sectioned using a vibratome (Leica, VT1200S) in high sucrose cutting solution, and immediately transferred to an incubation chamber containing oxygenated (95% O_2_ and 5% CO_2_) artificial cerebrospinal fluid (ACSF), consisting of 124 mM NaCl, 2.5 mM KCl, 2 mM CaCl_2_, 1 mM MgSO_4_, 25 mM NaHCO_3_, 1 mM NaH_2_PO_4_ and 10 mM glucose. Slices were allowed to recover in ACSF equilibrated with 95% O_2_ and 5% CO_2_ for at least 1 hour at room temperature.

Patch-clamp recordings on PrL layer II/III pyramidal neurons were performed using an upright microscope (BX51W, Olympus, USA) equipped with differential interference contrast (DIC) optics and digitized at 10 kHz using an Axopatch 200B amplifier (Molecular Devices, CA). Paired pulse ratio (PPR) and evoked excitatory postsynaptic current (EPSC) input/output curves were recorded with the K-gluconate intracellular solution and ACSF extracellular solution supplemented with picrotoxin (100 μM). PPRs were recorded with interstimulus intervals of 35, 50, 75, 100, and 150 ms. The average of five sweeps at each interval was calculated. The amplitude of an evoked EPSC was measured over five stimuli of different intensities ranging from 3 V to 7 V. In addition, miniature excitatory postsynaptic currents (mEPSCs) were also recorded in the presence of 1 µM tetrodotoxin and 100 μM picrotoxin. The amplitude and frequency of mEPSCs were calculated from a 3-min recording. Access resistance was 15-30 MΩ and was monitored throughout the experiment. Data were collected and analyzed using Clamper 10.2 and Clampfit 10.2 (Molecular Devices).

**AAV injection**

Mice were anesthetized with isoflurane and were randomly assigned to experimental groups. Virus injection was performed at the following coordinates relative to bregma: anteroposterior (AP) = 1.7 mm; mediolateral (ML) = ± 0.3 mm; dorsoventral (DV) = −2.7 mm. Viral suspension in a volume of 1.0 µl was injected into the mPFC using a microinjector at a rate of 0.2 µl/min. To prevent reflux, the needle was slowly withdrawn 10 minutes after injection. After behavioral tests, the accuracy of the injections was checked in each mouse. COP1 expression in the mPFC was confirmed by Western blot. Only mice with accurate mPFC injections were included in the analysis.

**Determination of the estrous cycle**

The estrous cycle was determined as described in our previous study^9^. Briefly, the stages were monitored daily by microscopic examination of vaginal smears. Mice with two consecutive 5-day estrous cycles were used.

References

1. Tan Z, Li Y, Guan Y, Iqbal J, Wang C, Yan R, et al. Klotho Regulated by Estrogen Plays a Key Role in Sex Differences in Stress Resilience in Rats. Int J Mol Sci. 2023;24:1206.

2. Sungur AO, Schwarting RK, Wohr M. Early communication deficits in the Shank1 knockout mouse model for autism spectrum disorder: Developmental aspects and effects of social context. Autism Res. 2016;9:696-09.

3. Gilbert J, O'Connor M, Templet S, Moghaddam M, Di Via Ioschpe A, Sinclair A, et al. NEXMIF/KIDLIA Knock-out Mouse Demonstrates Autism-Like Behaviors, Memory Deficits, and Impairments in Synapse Formation and Function. J Neurosci. 2020;40:237-54.

4. Deacon RM. Assessing nest building in mice. Nat Protoc. 2006;1:1117-19.

5. Zhuang PC, Tan ZN, Jia ZY, Wang B, Grady JJ, Ma XM. Treadmill Exercise Reverses Depression Model-Induced Alteration of Dendritic Spines in the Brain Areas of Mood Circuit. Front Behav Neurosci. 2019;13:93.

6. Kiraly DD, Ma XM, Mazzone CM, Xin X, Mains RE, Eipper BA. Behavioral and morphological responses to cocaine require kalirin7. Biol Psychiatry. 2010;68:249-55.

7. Nakayama AY, Harms MB, Luo L. Small GTPases Rac and Rho in the maintenance of dendritic spines and branches in hippocampal pyramidal neurons. J Neurosci. 2000;20:5329-38.

8. Ma XM, Huang J, Wang Y, Eipper BA, Mains RE. Kalirin, a multifunctional Rho guanine nucleotide exchange factor, is necessary for maintenance of hippocampal pyramidal neuron dendrites and dendritic spines. J Neurosci. 2003;23:10593-603.

9. Iqbal J, Tan ZN, Li MX, Chen HB, Ma B, Zhou X, et al. Estradiol Alters Hippocampal Gene Expression during the Estrous Cycle. Endocr Res. 2020:45:84-101.
