# Supplementary material for: Increased gene dosage of RFWD2 causes autistic-like behaviors and aberrant synaptic formation and function in mice
Source: Mol Psychiatry. 2024 Mar 19;29(8):2496–509. doi: 10.1038/s41380-024-02515-7 (PMC11412905; doi:10.1038/s41380-024-02515-7)
Supplement: Supplementary file 2 — Supplementary Figures [file 41380_2024_2515_MOESM2_ESM.docx]

**Increased gene dosage of *RFWD2* causes autistic-like behaviors and aberrant synaptic formation and function in mice**

Yong-Xia Li^1^, Zhi-Nei Tan^1^, Xu-Hui Li^2^, Boyu Ma^3^, Frank Adu Nti^1^, Xiao-Qiang Lv^1^, Zhen-Jun Tian^4^, Riqiang Yan^5^, Heng-Ye Man^6^*, Xin-Ming Ma^5^*

^1^College of Life Sciences, Shaanxi Normal University, Xi’an, China

^2^Center for Neuron and Disease, Frontier Institutes of Science and Technology, Xi’an Jiaotong University, Xi’an, China

^3^Department of Oral and Maxillofacial Surgery, University of Alabama at Birmingham, Birmingham, AL, United States

^4^Institute of Sports Biology, College of Physical Education, Shaanxi Normal University, Xi'an, China

^5^Department of Neuroscience, University of Connecticut Health, Farmington, CT, United States

^6^Department of Biology, Boston University, Boston, Massachusetts, United States

**Summary of results from six supplementary figures (SF):**

1. *Rfwd2*^flox/flox^ and *syn1-cre* mice showed normal communication, normal social behavior, and normal spatial learning and memory.
2. *Rfwd2*+/- *f*emale mice had subtle deficits in social communication and spatial memory compared to WT female littermates, but were unaffected in anxiety-like, repetitive, and social behaviors.
3. EVT5 expression in the mPFC did not alter social behavior in WT male mice compared to male WT control mice expressing GFP only.

SF. 1. *Rfwd2*^flox/flox^ mice and *syn1-cre* mice have the same genetic background and show normal behaviors in the open field and elevated plus maze tests.

SF. 2**.** *Rfwd2*^flox/flox^ mice and *syn1-cre* mice show normal communication in the ultrasonic vocalization test.

SF. 3*. Rfwd2*^flox/flox^ mice and *syn1-cre* mice show normal social behaviors in the 3-chamber test.

SF. 4*. Rfwd2*^flox/flox^ mice and *syn1-cre* mice show normal spatial learning and memory in the Barnes maze test.

SF. 5*. Rfwd2*+/- female mice show normal anxiety-like behavior, normal social behavior, and subtle deficits in social communication and spatial memory compared to WT female littermates.

SF. 6*.* Expression of ETV5 in the mPFC of WT male mice did not alter behaviors**.**

**
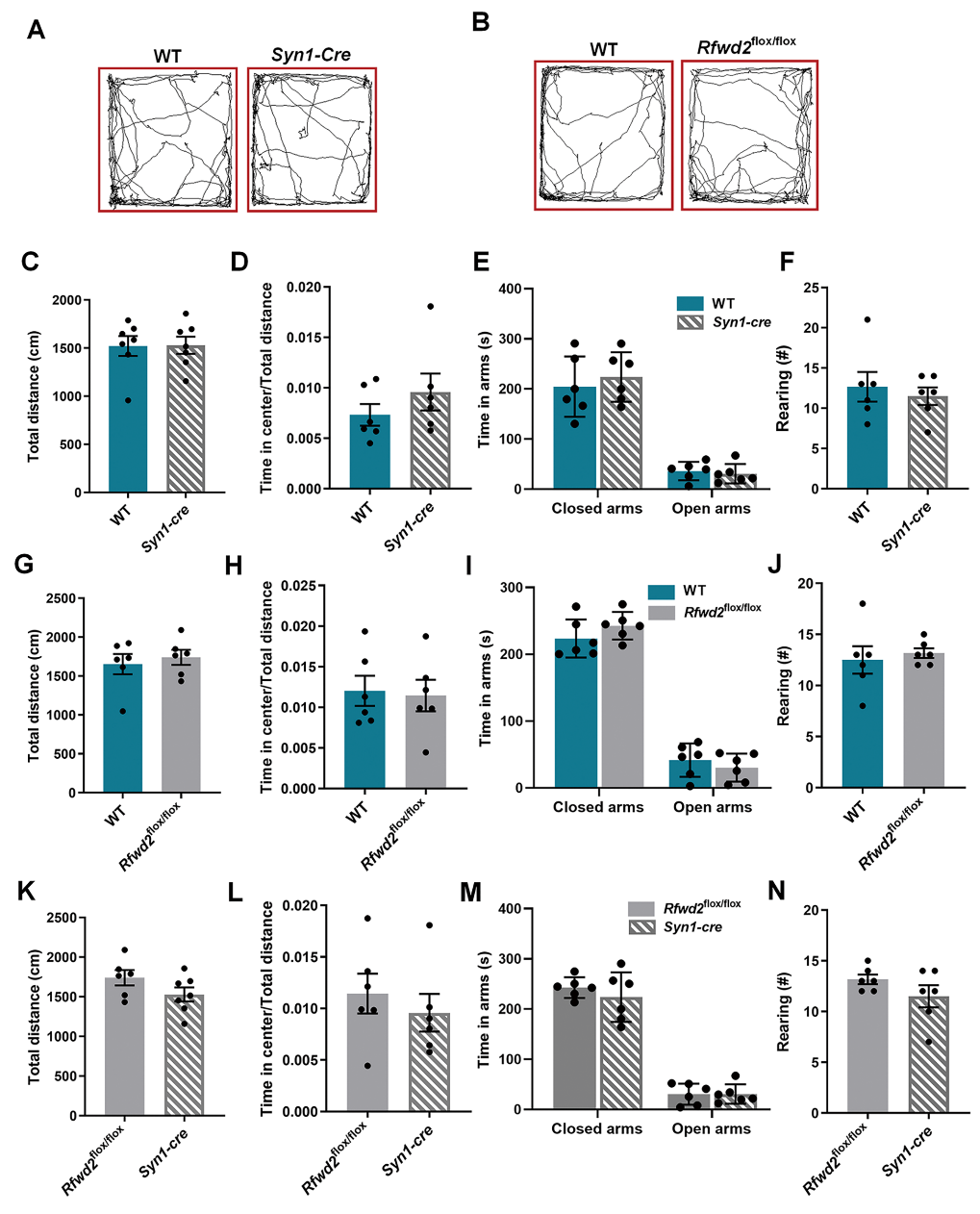
**

**Supplementary Figure 1. *Rfwd2*^flox/flox^ mice and *syn1-cre* mice have the same genetic background and show normal behaviors in the open field test (OFT) and the elevated plus maze** (**EPM).** (**A**-**B)** Representative traces in the OFT. There were no significant differences in locomotion (**C, G, K**), time spent in the center of the open field (**D, H, L**), the time spent in the closed arms and open arms (**E, I, M**), or rearing (**F, J, N**) between *syn1-cre* mice and their WT littermate controls (**C**-**F**), between *Rfwd2*^flox/flox^ mice and their WT littermate controls (**G**-**J**), or between *Rfwd2*^flox/flox^ mice and *syn1-cre* mice (**K**-**N**). Data are expressed as mean ± SEM, n=6.


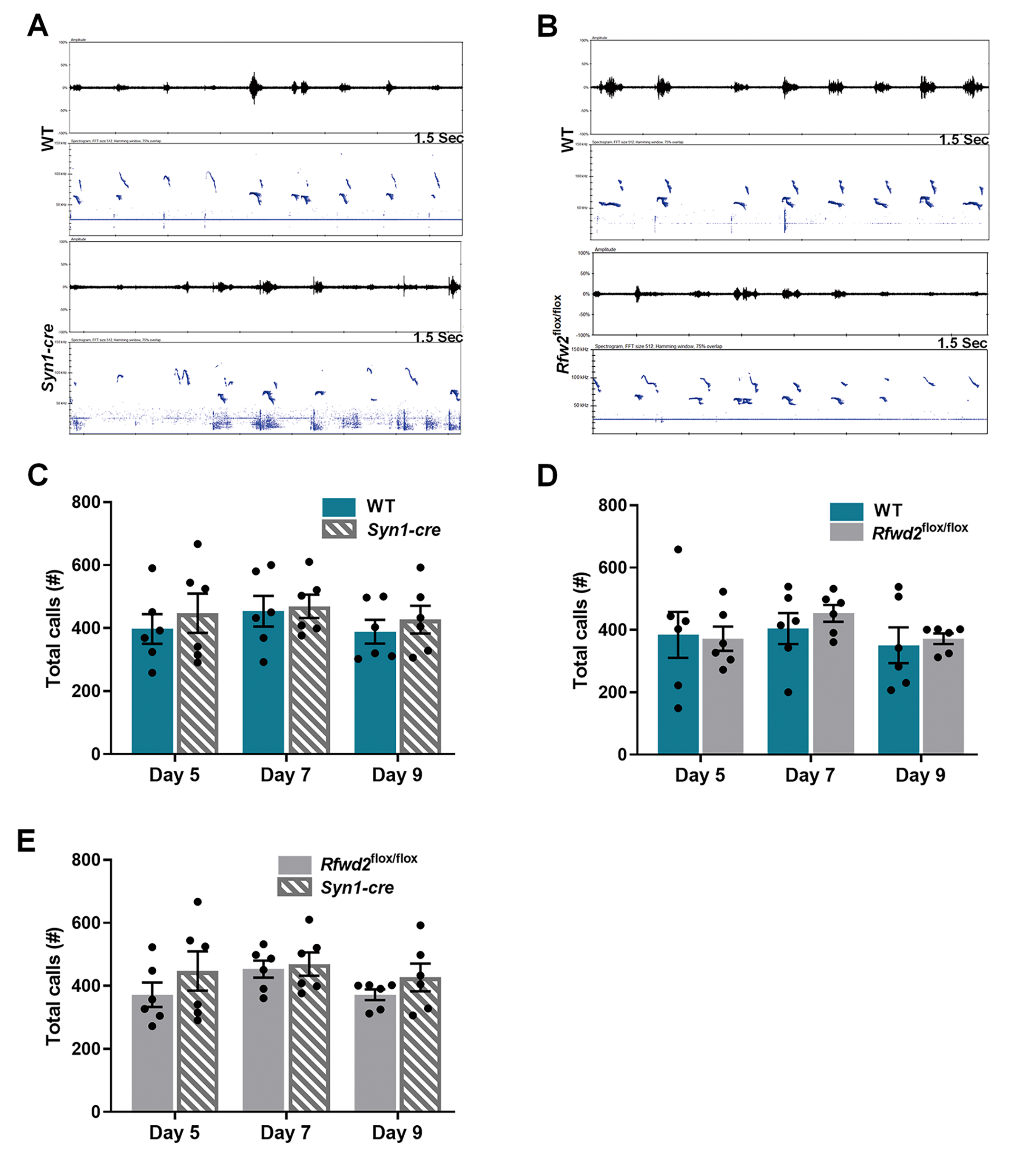


**Supplementary Figure 2. *Rfwd2*^flox/flox^ mice and *syn1-cre* mice show normal communication in the ultrasond vocalization test.** (**A**, **B**) Representative vocalizations from P7 recordings. Top, oscillogram signals. Bottom, corresponding spectrogram signals; all are 1.5 s long. There were no differences in the total number of calls during the 5-min recording period at any of the developmental time points (P5, P7, and P9) between *syn1-cre* mice and their WT littermate controls (**C**), between *Rfwd2*^flox/flox^ mice and their WT littermate controls (**D**), or between *Rfwd2*^flox/flox^ mice and *syn1-cre* mice (**E**). Data are presented as mean ± SEM, n=6.


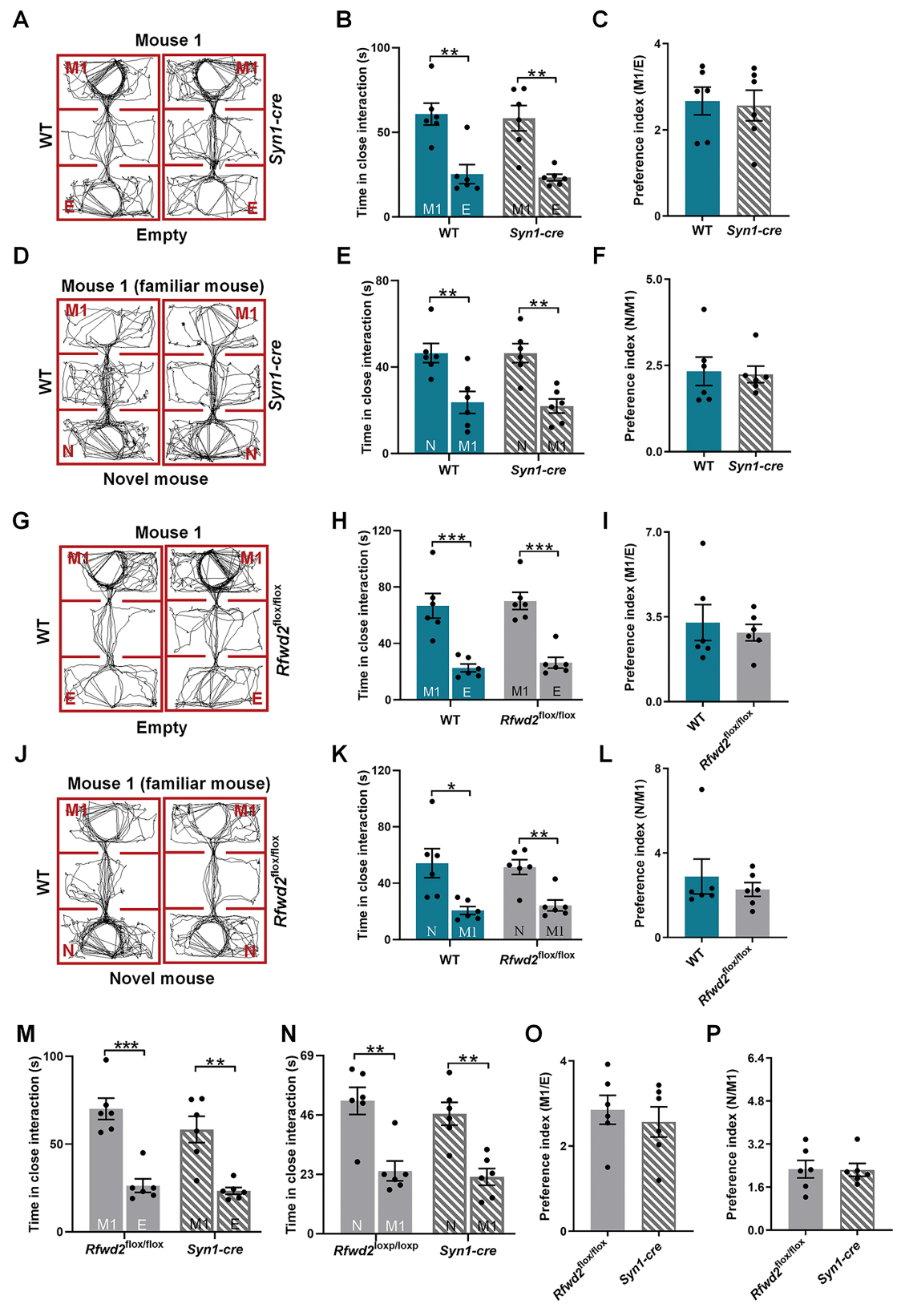


**Supplementary Figure 3. *Rfwd2*^flox/flox^ mice and *Syn1-cre* mice show normal social behaviors.** Representative traces in the sociability test for *Syn1-cre* (**A, D**) and *Rfwd2*^flox/flox^ mice (**G, J**). *Syn1-cre* mice (**B**-**C**) and *Rfwd2*^flox/flox^ mice (**H**-**I**) spent more time in the chamber containing the target (mouse 1, M1) than with the non-social target (empty, E) in the other chamber, respectively. (**C**, **I**) *Rfwd2*^flox/flox^ mice (**C**) and *syn1-cre* animals (**I**) showed no significant differences in preference index for the M1 mouse (MI/E) compared to their WT littermates, respectively, during the sociability test. Representative traces of a cre mouse (**D**) and a *Rfwd2*^flox/flox^ mouse (**J**) in the social novelty test. *Syn1-cre* mice (**D**, **E**) and *Rfwd2*^flox/flox^ mice (**J**, **K**) spent more time in the lower chamber with an unfamiliar mouse (Novel mouse, N) than in the top chamber with a familiar target (M1). Neither *Rfwd2*^flox/flox^ mice (**C**) nor *syn1-cre* animals (**I**) showed any significant difference in preference index for the novel mouse (N/M1) compared to their WT littermate controls, respectively, during the social novelty test. *Rfwd2*^flox/flox^ mice and *syn1-cre* mice spent more time with the M1 mouse than with the E (empty chamber, **M**), and with the N than with the M1 mouse (**N**) during the sociability and social novelty tests, respectively. There were no significant differences in the preference index for MI/E (**O**) or N/M1 (**P**) between *Rfwd2*^flox/flox^ mice and *syn1-cre* mice. Data are presented as mean ± SEM. n=6, **p*<0.05; ***p*<0.01, ****p*<0.001.


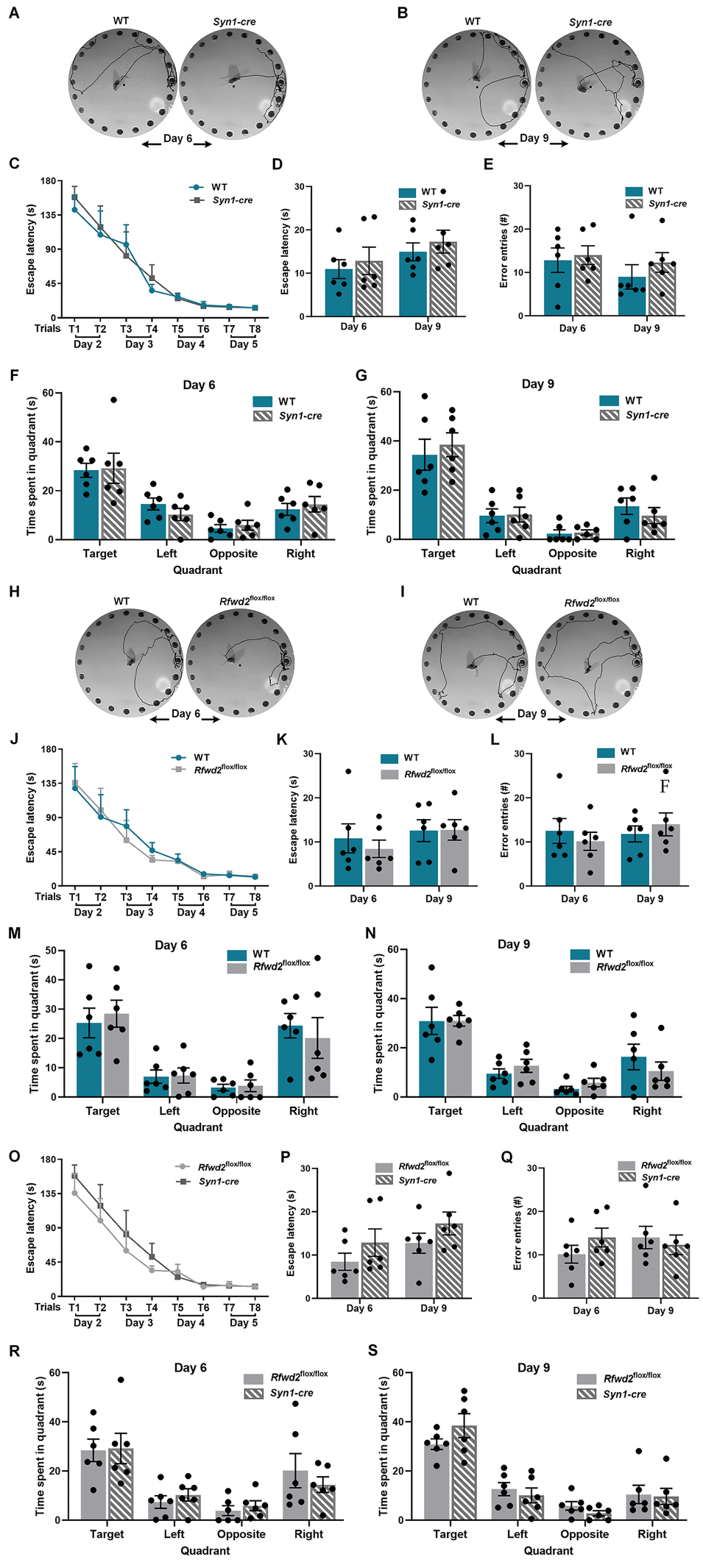


**Supplementary Figure 4. *Rfwd2*^flox/flox^ mice and *syn1-cre* mice show normal spatial learning and memory in the Barnes maze test.** Representative traces from a WT mouse and a *syn1-cre* mouse (**A, B**), as well as *Rfwd2*^+/-^ mice and WT littermates **(H, I),** during probe trials on days 6 and 9. (**C**, **J**, **O**) Escape latency on days 2-5 during training. Latency to locate the target hole during probe trials on day 6 or 9 was similar between *syn1-cre* mice and their WT littermates (**D**), *Rfwd2*^flox/flox^ mice and their WT littermates (**K**), and *Rfwd2*^flox/flox^ mice and *syn1-cre* mice (**P**). There were no significant differences in the number of errors made to reach the target hole during probe trials on day 6 or 9 between *syn1-cre* mice and their WT littermate controls (**E**), *Rfwd2*^flox/flox^ mice and their WT littermate controls (**L**), or *Rfwd2*^flox/flox^ mice and syn1-cre mice (**Q**). The time spent in the target quadrant during probe trials on day 6 or 9 was similar between *syn1-cre* mice and their WT littermate controls (**F**, **G**), *Rfwd2*^flox/flox^ mice and their WT littermate controls (**M**, **N**), and *Rfwd2*^flox/flox^ mice and *syn1-cre* mice (**R**, **S**) during probe trials on days 6 and 9. Data are presented as mean ± SEM, n=6.

**
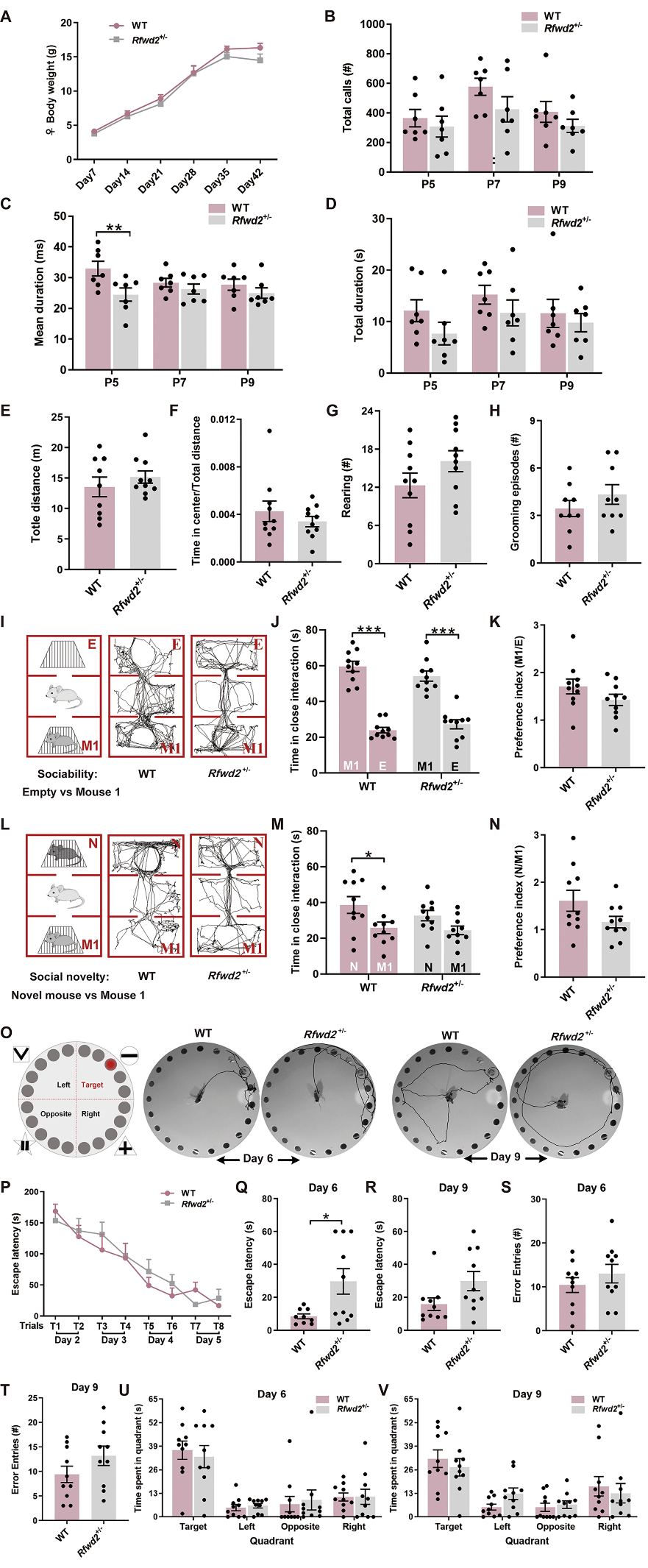
**

**Supplementary Figure 5. *Rfwd2*+/- female mice show normal anxiety-like behavior, normal social behavior, and subtle deficits in social communication and spatial memory. A.** Body weight of *Rfwd2*+/- female mice and WT female littermates during postnatal development. **B-C.** Five-minute ultrasonic vocalization recordings of *Rfwd2*^+/-^ and WT female littermates. The total number of calls (**B**), mean call duration (**C**) and total call duration (**D**) of *Rfwd2*^+/-^ and WT female littermates on postnatal (P) days 5, 7, and 9. Locomotion (**E**), time spent in the center of the open field (**F**), and the number of rearing episodes (**G**) and grooming episodes (**H**) in the open field test. **I**. Traces of animal track paths in the sociability test for *Rfwd2*^+/-^ and WT female mice. **J.** Interaction time spent with a novel mouse 1 (M1) and empty chamber (E). **K.** The preference index (M1/E ratio) during the sociability session. **L**. Traces of animal in the social novelty test for *Rfwd2*^+/-^female mice and WT female littermates. **M.** Interaction time spent with a novel mouse (N) and a familiar mouse (M1). **N.** The preference index (N/M1 ratio) during the sociability session. **O.** Tracking paths on days 6 and 9 in *Rfwd2*^+/-^ female mice and WT littermates in the Barnes maze test. **P.** Escape latency to the target hole on days 2-5 during the training trials. **Q.** Escape latency to the target hole on day 6. **R.** Escape latency to the target hole on day 9. **S.** Number of errors made in finding the target hole on day 6. **T.** Number of errors made in finding the target hole on day 9. **U.** The time spent in four quadrants on day 6. **V**. The time spent in four quadrants on day 9. A-D, P, two-way repeated measures ANOVA followed by Sidak’s test, J, M, two-way ANOVA followed by Bonferroni’s test, others with two-population student’s *t-test*. Data are presented as mean ± SEM. **p*<0.05; ****p*<0.001, n=10.


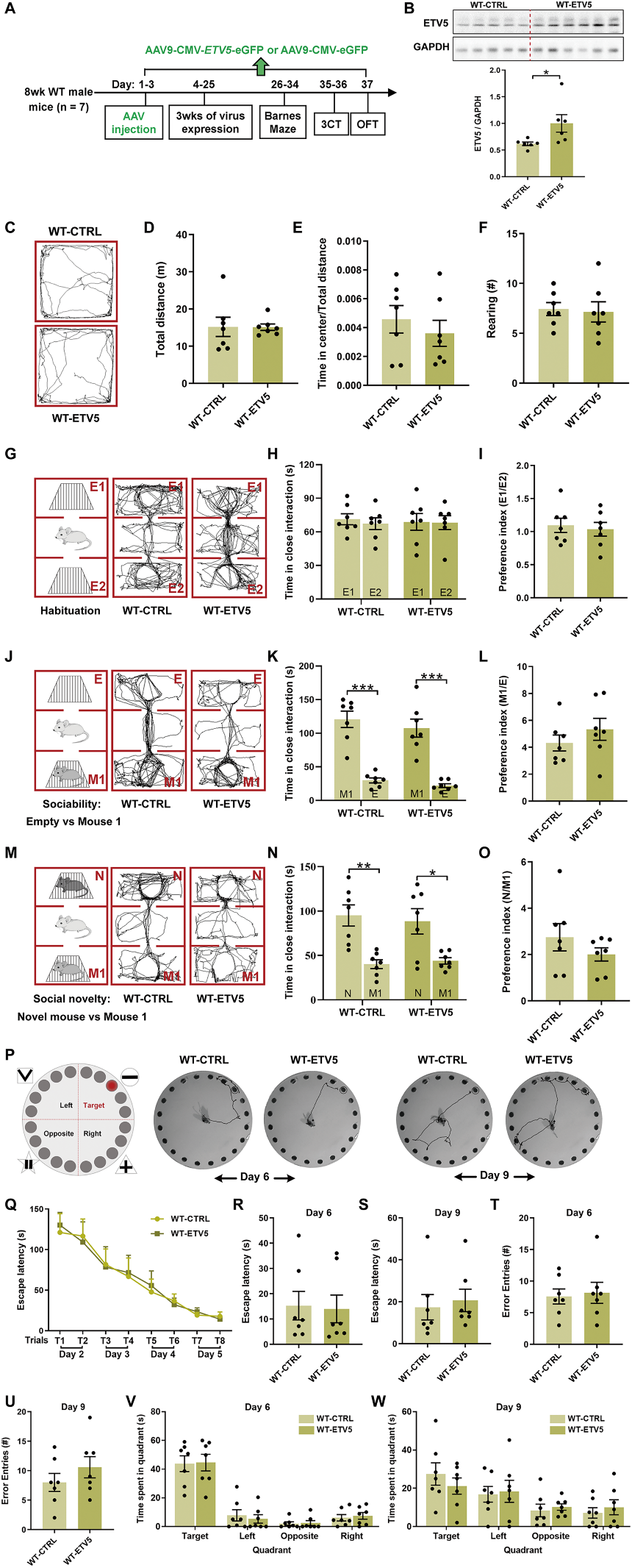


**Supplementary Figure 6. Expression of ETV5 in the mPFC of WT male mice does not alter behavior. A.** Experimental design. Stereotaxic bilateral injection of the AAV9-CMV-*ETV5*-eGFP (WT-ETV5) or empty vector AAV9-CMV-eGFP (WT-CTRL) into the mPFC of WT male mice (n=7). 3CT, 3-chamber test; OPT, open field test. **B.** ETV5 protein levels in the mPFC of WT-ETV5 and WT-CTRL mice. **C-F**: OFT. **C.** Representative traces in the open field of the OFT from a WT-ETV5 mouse and a WT-CTRL mouse. **D-F:** Locomotion (**D**), the time spent in the center of the open field (**E**) and rearing numbers (**F**) in the OFT. **G-O**: Three-chamber test. **G**. Tracking paths in the habituation session for a WT-ETV5 mouse and a WT-CTRL mouse. **H.** The interaction time spent in empty 1 cage (E1) and empty 2 cage (E2). **I.** Preference index (E1/E2 ratio) during the sociability session. **J**. Traces of animals in the sociability test for WT-ETV5 and WT-CTRL mice. **K.** The interaction time spent with a novel mouse 1 (M1) and in an empty (E) holding cell. **L.** The preference index (M1/E ratio) during the sociability session. **M**. Tracking paths in the social novelty test for WT-ETV5 and WT-CTRL mice. **N.** The interaction time spent with a novel mouse (N) and a familiar mouse (M1). **O.** The preference index (N/M1 ratio) during the sociability session. **P-W**: Barnes maze test. **P.** Traces of animal tracks on days 6 and 9 during the probe trials in WT-ETV5 and WT-CTRL mice in the Barnes maze test. **Q.** Latency to locate the target hole on days 2-5 during the training. **R.** Latency to locate the target hole on day 6. **S.** Latency to the target hole on day 9. **T.** The number of errors made in finding the target hole on day 6. **U.** The number of errors made in finding the target hole on day 9. **V.** Time spent in four quadrants on day 6. **W.** Time spent in four quadrants on day 9. H, K, N, Two-way ANOVA followed by Bonferroni’s test. Q, Two-way repeated measures ANOVA followed by Sidak’s test, others with two-population Student’s t-test. Data are presented as mean ± SEM. **p*<0.05; ***p*<0.01, ****p*<0.001, n=6.
