# Supplementary material for: Increased gene dosage of RFWD2 causes autistic-like behaviors and aberrant synaptic formation and function in mice
Source: Mol Psychiatry. 2024 Mar 19;29(8):2496–509. doi: 10.1038/s41380-024-02515-7 (PMC11412905; doi:10.1038/s41380-024-02515-7)
Supplement: Supplementary file 3 — Supplementary Table 1 [file 41380_2024_2515_MOESM3_ESM.docx]

| **Two-way repeated-measure ANOVA** | | | | | | | | |  |
| --- | --- | --- | --- | --- | --- | --- | --- | --- | --- |
|  |  |  |  |  |  |  |  |  |  |
| Fig. 1 | Parameter | Assume spherity? | genotype × time | | genotype | | time | |  |
| Fig. 1J | Body weight | No, with Geisser-Greenhouse correction | *F*_8,80_ = 1.86 | *p* = 0.08 | *F*_1,10_ = 1.46 | *p* = 0.25 | *F*_2.85,28.5_ = 846.30 | *p* < 0.001 |  |
|  | | | | | | | | |  |
| Fig. 2 | Parameter | Assume spherity? | genotype × time | | genotype | | time | |  |
| Fig. 2B | Total calls | Yes, *p* = 0.17 | *F*_2,36_ = 1.74 | *p* = 0.19 | *F*_1,18_ = 135.80 | *p* < 0.001 | *F*_2.36_ = 1.97 | *p* = 0.16 |  |
| Fig. 2C | Mean duration | Yes, *p* = 0.64 | *F*_2,36_ = 1.95 | *p* = 0.16 | *F*_1,18_ = 18.97 | *p* < 0.001 | *F*_2,36_ = 1.27 | *p* = 0.29 |  |
| Fig. 2D | Total duration | Yes, *p* = 0.20 | *F*_2,36_ = 1.85 | *p* = 0.17 | *F*_1,18_ = 96.33 | *p* < 0.001 | *F*_2,36_ = 2.64 | *p* = 0.09 |  |
| Fig. 2E | Number of calls (P5) | Yes, *p* = 0.52 | *F*_4,64_ = 1.63 | *p* = 0.18 | *F*_1,16_ = 10.06 | *p* < 0.001 | *F*_4,64_ = 3.25 | *p* < 0.05 |  |
| Fig. 2F | Number of calls (P7) | Yes, *p* = 0.50 | *F*_4,64_ = 0.22 | *p* = 0.93 | *F*_1,16_ = 82.24 | *p* < 0.001 | *F*_4,64_ = 1.52 | *p* = 0.21 |  |
| Fig. 2G | Number of calls (P9) | Yes, *p* = 0.12 | *F*_4,64_ = 0.63 | *p* = 0.64 | *F*_1,16_ = 36.75 | *p* < 0.001 | *F*_4,64_ = 1.63 | *p* = 0.18 |  |
|  | | | | | | | | |  |
| Fig. 3 | Parameter | Assume spherity? | genotype × time | | genotype | | time | |  |
| Fig. 3I | Escape latency | No, with Geisser-Greenhouse correction | *F*_7,119_ = 0.46 | *p* = 0.86 | *F*_1,17_ = 0.34 | *p* = 0.57 | *F*_3.88,65.88_ = 9.10 | *p* < 0.001 |  |
|  | | | | | | | | |  |
| Fig. 4 | Parameter | Assume spherity? | genotype × stimulation intensity | | genotype | | stimulation intensity | |  |
| Fig. 4M | EPSCs Amplitude | No, with Geisser-Greenhouse correction | *F*_4,71_ = 3.27 | *p* < 0.05 | *F*_1,18_ = 8.56 | *p* < 0.01 | *F*_1.76,31.23_ = 87.70 | *p* < 0.001 |  |
| Fig. 4O | Paired-pulse Ratio | No, with Geisser-Greenhouse correction | *F*_4,79_ = 3.00 | *p* < 0.05 | *F*_1,20_ = 2.20 | *p* = 0.16 | *F*_3.20,63.20_ = 7.71 | *p* < 0.001 |  |
|  | | | | | | | | |  |
| Fig. 5 | Parameter | Assume spherity? | genotype × stimulation intensity | | genotype | | stimulation intensity | |  |
| Fig. 5B | Mean duration | Yes, *p* = 0.23 | *F*_2,24_ = 1.62 | *p* = 0.22 | *F*_1,12_ = 9.91 | *p* < 0.01 | *F*_2,24_ = 0.75 | *p* = 0.49 |  |
| Fig. 5S | EPSCs Amplitude | No, with Geisser-Greenhouse correction | *F*_4,76_ = 3.48 | *p* < 0.05 | *F*_1,19_ = 2.11 | *p* = 0.16 | *F*_1.66,31.52_ = 128.50 | *p* < 0.001 |  |
|  | | | | | | | | |  |
| Fig. 6 | Parameter | Assume spherity? | treatment × position | | treatment | | position | |  |
| Fig. 6Q | Escape latency | No, with Geisser-Greenhouse correction | *F*_7,70_ = 0.28 | *p* = 0.98 | *F*_1,10_ = 0.12 | *p* = 0.73 | *F*_2.98,29.8_ = 21.04 | *p* < 0.001 |  |
|  |  |  |  |  |  |  |  |  |  |
| SF. 5 | Parameter | Assume spherity? | genotype × time | | genotype | | time | |  |
| SF. 5A | Body weight | No, with Geisser-Greenhouse correction | *F*_5,50_ = 0.95 | *p* = 0.46 | *F*_1,10_ = 1.07 | *p* = 0.32 | *F*_2.40,24.0_ = 21.04 | *p* < 0.001 |  |
| SF. 5B | Total calls | Yes, *p* = 0.50 | *F*_2,24_ = 0.15 | *p* = 0.86 | *F*_1,12_ = 5.71 | *p* = 0.03 | *F*_2,24_ = 1.16 | *p* = 0.33 |  |
| SF. 5C | Total duration | No, with Geisser-Greenhouse correction | *F*_2,24_ = 0.28 | *p* = 0.75 | *F*_1,12_ = 3.13 | *p* = 0.10 | *F*_1.40,16.84_ = 3.91 | *p* = 0.05 |  |
| SF. 5P | Escape latency | No, with Geisser-Greenhouse correction | *F*_7,126_ = 1.15 | *p* = 0.33 | *F*_1,18_ = 0.78 | *p* = 0.39 | *F*_3.76,67.70_ = 22.69 | *p* < 0.001 |  |
|  | | | | | | | | |  |
| SF. 6 | Parameter | Assume spherity? | treatment × position | | treatment | | position | |  |
| SF. 6Q | Escape latency | No, with Geisser-Greenhouse correction | *F*_7,84_ = 0.06 | *p* = 0.10 | *F*_1,12_ = 0.02 | *p* = 0.89 | *F*_3.63,43.58_ = 10.88 | *p* < 0.001 |  |
|  | | | | | | | | |  |
| Violation of Sphericity, with Geisser-Greenhouse correction. Two-way repeated-measure ANOVA, followed by Sidak’s test. | | | | | | | | |  |
|  |  |  |  |  |  |  |  |  |  |

| **Two-way ANOVA** | | | | | | | |  |
| --- | --- | --- | --- | --- | --- | --- | --- | --- |
|  |  |  |  |  |  |  |  |  |
| Fig. 2 | Parameter | genotype × position | | genotype | | position | |  |
| Fig. 2J | Time in close interaction (E1and E2) | *F*_1,36_ = 0.09 | *p* = 0.76 | *F*_1,36_ = 0.28 | *p* = 0.60 | *F*_1,36_ = 0.24 | *p* = 0.62 |  |
| Fig. 2N | Time in close interaction (M1and E) | *F*_1,36_ = 0.0003 | *p* = 0.98 | *F*_1,36_ = 0.14 | *p* = 0.71 | *F*_1,36_ = 43.95 | *p* < 0.001 |  |
| Fig. 2R | Time in close interaction (N and M1) | *F* _1, 36_ = 4.56 | *p* < 0.05 | *F*_1,36_ = 0.0005 | *p* = 0.98 | *F*_1,36_ = 3.01 | *p* = 0.09 |  |
|  | | | | | | | |  |
| Fig. 6 | Parameter | treatment × position | | treatment | | position | |  |
| Fig. 6H | Time in close interaction (E1and E2) | *F*_1,20_ = 0.05 | *p* = 0.82 | *F*_1,20_ = 0.08 | *p* = 0.78 | *F*_1,20_ = 0.41 | *p* = 0.53 |  |
| Fig. 6K | Time in close interaction (M1and E) | *F*_1,20_ = 0.02 | *p* = 0.89 | *F*_1,20_ = 1.14 | *p* = 0.30 | *F*_1,20_ = 39.09 | *p* < 0.001 |  |
| Fig. 6N | Time in close interaction (N and M1) | *F*_1,20_ = 0.02 | *p* = 0.09 | *F*_1,20_ = 0.80 | *p* = 0.38 | *F*_1,20_ = 5.64 | *p* < 0.05 |  |
|  |  |  |  |  |  |  |  |  |
| SF. 5 | Parameter | genotype × position | | genotype | | position | |  |
| SF. 5J | Time in close interaction (M1and E) | *F*_1,36_ = 3.00 | *p* = 0.09 | *F*_1,36_ = 0.18 | *p* = 0.68 | *F*_1,36_ = 156.20 | *p* < 0.001 |  |
| SF. 5M | Time in close interaction (N and M1) | *F*_1,36_ = 0.44 | *p* = 0.51 | *F*_1,36_ = 1.14 | *p* = 0.29 | *F*_1,36_ = 9.46 | *p* < 0.01 |  |
|  | | | | | | | |  |
| SF. 6 | Parameter | treatment × position | | treatment | | position | |  |
| SF. 6H | Time in close interaction (E1and E2) | *F*_1,24_ = 0.08 | *p* = 0.78 | *F*_1,24_ = 0.01 | *p* = 0.91 | *F*_1,24_ = 0.14 | *p* = 0.17 |  |
| SF. 6K | Time in close interaction (M1and E) | *F*_1,24_ = 0.08 | *p* = 0.78 | *F*_1,24_ = 1.23 | *p* = 0.28 | *F*_1,24_ = 88.88 | *p* < 0.001 |  |
| SF. 6N | Time in close interaction (N and M1) | *F*_1,24_ = 0.27 | *p* = 0.61 | *F*_1,24_ = 0.02 | *p* = 0.89 | *F*_1,24_ = 25.60 | *p* < 0.001 |  |
|  | | | | | | | |  |
| Two-way ANOVA followed by Bonferroni’s test | | | | | | | |  |
|  |  |  |  |  |  |  |  |  |

| **Two-population student’s *t* test** | | | | | |  |
| --- | --- | --- | --- | --- | --- | --- |
|  |  |  |  |  |  |  |
| Fig. 1 | Parameter | Std. Error of Mean | | *t* Value | *p* Value |  |
|  |  | WT | *Rfwd2*^+/-^ |  |  |  |
| Fig. 1E | RFWD2 / GAPDH (NAc) | 1.00 ± 0.06 | 3.60 ± 0.37 | *t*_(12)_ = 6.92 | *p* < 0.0001 |  |
|  | RFWD2 / GAPDH (mPFC) | 1.00 ± 0.02 | 3.85 ± 0.17 | *t*_(12)_ = 16.52 | *p* < 0.0001 |  |
|  | RFWD2 / GAPDH (Hip) | 1.00 ± 0.24 | 4.6 ± 0.13 | *t*_(12)_ = 13.13 | *p* < 0.0001 |  |
|  | RFWD2 / GAPDH (Hyp) | 1.00 ± 0.07 | 4.8 ± 0.23 | *t*_(12)_ = 13.13 | *p* < 0.0001 |  |
| Fig. 1G | PL thickness | 0.80 ± 0.01 | 0.82 ± 0.01 | *t*_(9)_ = 1.17 | *p* = 0.27 |  |
| Fig. 1I | Number of cells (DG) | 220.60 ± 8.36 | 222.60 ± 7.50 | *t*_(8)_ = 0.18 | *p* = 0.86 |  |
|  | Number of cells (CA3) | 172.40 ± 5.31 | 173.40 ± 6.06 | *t*_(8)_ = 0.12 | *p* = 0.90 |  |
|  | Number of cells (CA1) | 183.40 ± 6.50 | 174.20 ± 4.36 | *t*_(8)_ = 1.18 | *p* = 0.27 |  |
|  | | | | | |  |
| Fig. 2 | Parameter | Std. Error of Mean | | *t* Value | *p* Value |  |
|  |  | WT | *Rfwd2*^+/-^ |  |  |  |
| Fig. 2K | Preference Index (E1/E2) | 1.25 ± 0.20 | 1.20 ± 0.19 | *t*_(18)_ = 0.19 | *p* = 0.85 |  |
| Fig. 2O | Preference Index (M1/E) | 13.47 ± 4.62 | 3.88 ± 0.95 | *t*_(17)_ = 2.14 | *p* = 0.047 |  |
| Fig. 2S | Preference Index (N/M1) | 2.92 ± 0.67 | 0.95 ± 0.17 | *t*_(18)_ = 2.88 | *p* = 0.01 |  |
|  | | | | | |  |
| Fig. 3 | Parameter | Std. Error of Mean | | *t* Value | *p* Value |  |
|  |  | WT | *Rfwd2*^+/-^ |  |  |  |
| Fig. 3B | Total distance (m) | 19.85 ± 1.93 | 23.00 ± 2.36 | *t*_(18)_ = 1.04 | *p* = 0.31 |  |
| Fig. 3C | Time in center/Total distance | 0.004 ± 0.0008 | 0.002 ± 0.0002 | *t*_(18)_ = 2.37 | *p* = 0.03 |  |
| Fig. 3D | Grooming episodes (#) | 2.80 ± 0.60 | 5.00 ± 0.73 | *t*_(17)_ = 2.37 | *p* = 0.03 |  |
| Fig. 3E | Rearing (#) | 12.10 ± 2.19 | 23.60 ± 2.85 | *t*_(18)_ = 3.20 | *p* = 0.005 |  |
| Fig. 3G | Nesting score | 3.30 ± 0.42 | 2.09 ± 0.25 | *t*_(18)_ = 2.47 | *p* = 0.02 |  |
| Fig. 3J | Escape latency (s), Day 6 | 7.81 ± 2.20 | 22.40 ± 4.97 | *t*_(18)_ = 2.69 | *p* = 0.015 |  |
| Fig. 3K | Error entries (#), Day 6 | 10.60 ± 1.54 | 21.20 ± 2.65 | *t*_(18)_ = 3.46 | *p* = 0.003 |  |
| Fig. 3L | Escape latency (s), Day 9 | 11.38 ± 3.78 | 29.64 ± 7.37 | *t*_(18)_ = 2.21 | *p* = 0.04 |  |
| Fig. 3M | Error entries (#), Day 9 | 9.20 ± 1.97 | 12.10 ± 2.55 | *t*_(18)_ = 0.90 | *p* = 0.38 |  |
|  | | | | | |  |
| Fig. 4 | Parameter | Std. Error of Mean | | *t* Value | *p* Value |  |
|  |  | WT | *Rfwd2*^+/-^ |  |  |  |
| Fig. 4B | Spine density / 10 μm | 5.13 ± 0.30 | 3.75 ± 0.31 | *t*_(10)_ = 3.18 | *p* = 0.01 |  |
| Fig. 4C | Mushroom spines | 2.17 ± 0.23 | 1.50 ± 0.16 | *t*_(10)_ = 2.39 | *p* = 0.04 |  |
| Fig. 4E | ETV5 / GAPDH | 1.00 ± 0.05 | 0.51 ± 0.03 | *t*_(12)_ = 8.98 | *p* < 0.0001 |  |
| Fig. 4F | PSD95 / GAPDH | 1.00 ± 0.06 | 0.89 ± 0.09 | *t*_(12)_ = 0.95 | *p* = 0.36 |  |
| Fig. 4G | Vgult 1 / GAPDH | 1.00 ± 0.02 | 0.91 ± 0.02 | *t*_(10)_ = 2.67 | *p* = 0.04 |  |
| Fig. 4H | GluN 2B / GAPDH | 1.00 ± 0.08 | 0.74 ± 0.08 | *t*_(12)_ = 2.22 | *p* = 0.047 |  |
| Fig. 4I | GluN 1 / GAPDH | 1.00 ± 0.09 | 0.92 ± 0.03 | *t*_(10)_ = 0.82 | *p* = 0.43 |  |
| Fig. 4J | GluA 2 / GAPDH | 1.00 ± 0.04 | 0.91 ± 0.02 | *t*_(10)_ = 1.85 | *p* = 0.09 |  |
| Fig. 4Q | mEPSC frequency (Hz) | 2.62 ± 0.24 | 1.94 ± 0.18 | *t*_(30)_ = 2.23 | *p* = 0.03 |  |
| Fig. 4R | mEPSC amplitude (pA) | 7.09 ± 0.41 | 6.08 ± 0.25 | *t*_(30)_ = 2.12 | *p* = 0.04 |  |
|  | | | | | |  |
| Fig. 5 | Parameter | Std. Error of Mean | | *t* Value | *p* Value |  |
|  |  | WT | *Rfwd2*^+/-^ |  |  |  |
| Fig. 5C | Escape latency (s), Day 6 | 8.46 ± 1.49 | 29.68 ± 7.73 | *t*_(17)_ = 2.56 | *p* = 0.02 |  |
| Fig. 5E | Spine density / 10 μm | 5.75 ± 0.53 | 4.00 ± 0.41 | *t*_(10)_ = 2.62 | *p* = 0.03 |  |
| Fig. 5F | Filopiodia density | 0.92 ± 0.30 | 1.75 ± 0.17 | *t*_(10)_ = 2.41 | *p* = 0.04 |  |
| Fig. 5H | RFWD2 / GAPDH (NAc) | 1.00 ± 0.11 | 3.60 ± 0.23 | *t*_(10)_ = 10.08 | *p* < 0.0001 |  |
| Fig. 5I | RFWD2 / GAPDH (Hip) | 1.00 ± 0.18 | 3.04 ± 0.12 | *t*_(10)_ = 16.4 | *p* < 0.0001 |  |
| Fig. 5J | RFWD2 / GAPDH (Hyp) | 1.00 ± 0.21 | 2.88 ± 0.12 | *t*_(10)_ = 7.75 | *p* < 0.0001 |  |
| Fig. 5K | RFWD2 / GAPDH (mPFC) | 1.00 ± 0.03 | 3.36 ± 0.09 | *t*_(10)_ = 24.07 | *p* < 0.0001 |  |
| Fig. 5L | GluN 2B / GAPDH | 1.00 ± 0.03 | 0.98 ± 0.02 | *t*_(10)_ = 0.44 | *p* = 0.67 |  |
| Fig. 5M | GluA 1 / GAPDH | 1.00 ± 0.06 | 0.83 ± 0.04 | *t*_(10)_ = 2.32 | *p* = 0.043 |  |
| Fig. 5N | ETV5 / GAPDH | 1.00 ± 0.10 | 0.69 ± 0.05 | *t*_(11)_ = 2.95 | *p* = 0.01 |  |
| Fig. 5O | Vgult 1 / GAPDH | 1.00 ± 0.06 | 0.99 ± 0.04 | *t*_(10)_ = 0.03 | *p* = 0.98 |  |
| Fig. 5P | GluA 2 / GAPDH | 1.00 ± 0.02 | 1.04 ± 0.07 | *t*_(10)_ = 0.58 | *p* = 0.58 |  |
| Fig. 5U | mEPSC frequency | 2.27 ± 0.31 | 1.57 ± 0.14 | *t*_(17)_ = 2.12 | *p* = 0.049 |  |
| Fig. 5V | mEPSC amplitude | 6.83 ± 0.65 | 6.03 ± 0.32 | *t*_(20)_ = 1.17 | *p* = 0.26 |  |
|  | | | | | |  |
| Fig. 6 | Parameter | Std. Error of Mean | | *t* Value | *p* Value |  |
|  |  | AAV-CTRL | AAV-ETV5 |  |  |  |
| Fig. 6D | Total distance (m) | 13.06 ± 0.59 | 14.61 ± 1.69 | *t*_(10)_ = 0.87 | *p* = 0.40 |  |
| Fig. 6E | Time in center/Total distance | 0.003 ± 0.0005 | 0.004 ± 0.0005 | *t*_(10)_ = 0.69 | *p* = 0.50 |  |
| Fig. 6F | Rearing (#) | 17.50 ± 2.51 | 13.33 ± 1.91 | *t*_(10)_ = 1.32 | *p* = 0.22 |  |
| Fig. 6I | Preference Index (E1/E2) | 1.00 ± 0.20 | 1.07 ± 0.29 | *t*_(10)_ = 0.19 | *p* = 0.85 |  |
| Fig. 6L | Preference Index (M1/E) | 2.71 ± 0.45 | 3.14 ± 0.44 | *t*_(10)_ = 0.68 | *p* = 0.51 |  |
| Fig. 6O | Preference Index (N/M1) | 1.05 ± 0.08 | 1.56 ± 0.16 | *t*_(9)_ = 2.98 | *p* = 0.02 |  |
| Fig. 6R | Escape latency (s), Day 6 | 21.48 ± 8.36 | 13.4 ± 1.85 | *t*_(10)_ = 0.94 | *p* = 0.37 |  |
| Fig. 6S | Escape latency (s), Day 9 | 28.50 ± 7.86 | 28.67 ± 7.73 | *t*_(10)_ = 0.01 | *p* = 0.99 |  |
| Fig. 6T | Error entries (#), Day 6 | 10.67 ± 1.05 | 8.67 ± 0.67 | *t*_(10)_ = 1.60 | *p* = 0.14 |  |
| Fig. 6U | Error entries (#), Day 9 | 10.67 ± 1.98 | 11.50 ± 2.96 | *t*_(10)_ = 0.23 | *p* = 0.82 |  |
